# Exploring the Chemical Properties and Medicinal Applications of Tetramethylthiocycloheptyne Sulfoximine Used in Strain-Promoted Azide–Alkyne Cycloaddition Reactions

**DOI:** 10.3390/ph16081155

**Published:** 2023-08-15

**Authors:** Matt Timmers, Andi Kipper, Raphael Frey, Stef Notermans, Maksym Voievudskyi, Claire Wilson, Nina Hentzen, Michael Ringle, Clara Bovino, Bernhard Stump, Cristianne J. F. Rijcken, Tina Vermonden, Ingrid Dijkgraaf, Rob Liskamp

**Affiliations:** 1 Cristal Therapeutics, 6229 EV Maastricht, The Netherlands; 2 Department of Pharmaceutics, Utrecht Institute for Pharmaceutic Sciences, Utrecht University, 3584 CG Utrecht, The Netherlands; 3 TBD Pharmatech, 50410 Tartu, Estoniamaksym.voievudskyi@tbdpharmatech.com (M.V.); 4 Lonza AG, Bioconjugates Development, Rottenstr., 3930 Visp, Switzerlandnina.hentzen@lonza.com (N.H.); michael.ringle@lonza.com (M.R.); bernhard.stump@lonza.com (B.S.); 5 Department of Biochemistry, Cardiovascular Research Institute Maastricht (CARIM), Maastricht University, 6229 ER Maastricht, The Netherlands; 6 School of Chemistry, University of Glasgow, Glasgow G12 8QQ, UK

**Keywords:** SPAAC, click chemistry, stability, bio-orthogonal chemistry, bioconjugation

## Abstract

The recently developed compound, tetramethylthiocycloheptyne sulfoximine (TMTHSI), has shown to be a promising strained alkyne for strain-promoted azide–alkyne cycloaddition (SPAAC), metal-free click chemistry. This research explores the properties of TMTHSI-based compounds via three aspects: (1) large-scale production, (2) unique stability in acidic conditions and its subsequent use in peptide synthesis, and (3) the functionalization of antibodies. Here, it is shown that (1) scale-up is achieved on a scale of up to 100 g. (2) TMTHSI is remarkably stable against TFA allowing for the site-specific functionalization of peptides on resin. Finally, (3) the functionalization of an antibody with a model payload is very efficient, with antibody conjugation demonstrating more beneficial features such as a high yield and limited hydrophobicity as compared to other alkyne reagent conjugates. These results illustrate the high potential of TMTHSI for diverse bioconjugation applications, with production already being GMP-compatible and a highly efficient conversion resulting in attractive costs of goods.

## 1. Introduction

Strain-promoted azide–alkyne cycloaddition (SPAAC) has become an important and versatile tool for applications involving the bio-orthogonal conjugation of biomolecular constructs in areas ranging from chemical and biological sciences to medical and material sciences. Initially, the copper-catalyzed azide–alkyne cycloaddition (CuAAC) opened up a plethora of possibilities [1,2], especially applications in biological settings which were limited due to the toxicity of residual copper. To broaden applications to medicinal chemistry, strain-promoted azide–alkyne cycloaddition (SPAAC) was developed [3]. The strain of alkyne functionality was induced by cyclization, thereby compromising its linearity and increasing the reactivity of alkynes. This development led to two widely used reagents: dibenzocyclooctyne (DBCO) [4] and bicyclononyne (BCN) [5]. These compounds have proven to be relevant in medicinal chemistry settings, with BCN even being applied in clinical trials of antibody–drug conjugates (ADCs) [6]. SPAAC is also used in the PEGylation of antibodies or liposomes, for example [7,8]. The size and hydrophobicity of the alkyne reagent is relevant, e.g., to tune the properties of the yielding bioconjugates in terms of aggregation behavior. Aggregation behavior can negatively influence pharmacokinetic properties of chemically modified proteins that act as biopharmaceutical drug candidates. The prospect of generating molecules with desired properties triggered the need to develop more reactive strained alkyne reagents, which are more hydrophilic, while maintaining the excellent stability and biorthogonality of DBCO or BCN.

**Figure 1:**
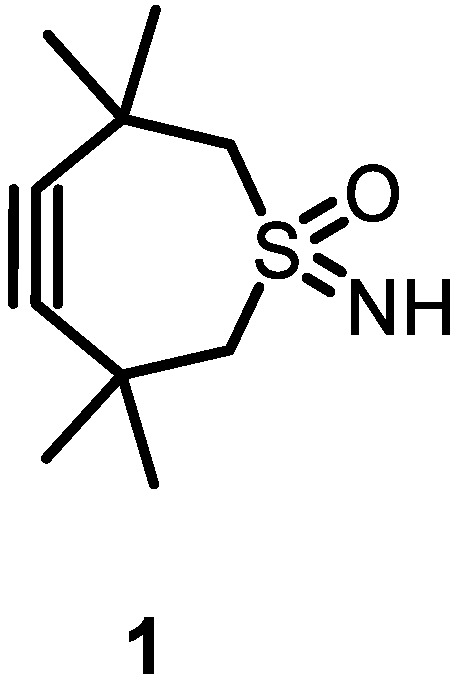
SPAAC reagent, TMTHSI.

In order to obtain a more reactive SPAAC reagent, tetramethylthiocycloheptyne sulfoximine (TMTHSI) was developed [9] (Figure 1). TMTHSI, marketed as “CliCr^®^”, has shown fast reactivity and limited hydrophobicity owing to its small size and hydrophilic sulfoximine [10]. While its synthesis and primary application has been reported, additional research is required for a full evaluation of the potential applications of this reagent. To this end, we discuss its industrial applicability from three aspects. First, the large-scale production is discussed to obtain insights into the scalability of the synthesis and possible improvements in cost effectiveness. Next, the chemical stability of relevant derivatives was studied to investigate the possible fields of application of this reagent. Lastly, antibody conjugation as a particularly relevant bioconjugation application was evaluated to provide insight into the behavior of the TMTHSI reagent.

## 2. Results and Discussion

### 2.1. Large-Scale Production of TMTHSI

**Scheme 1:**
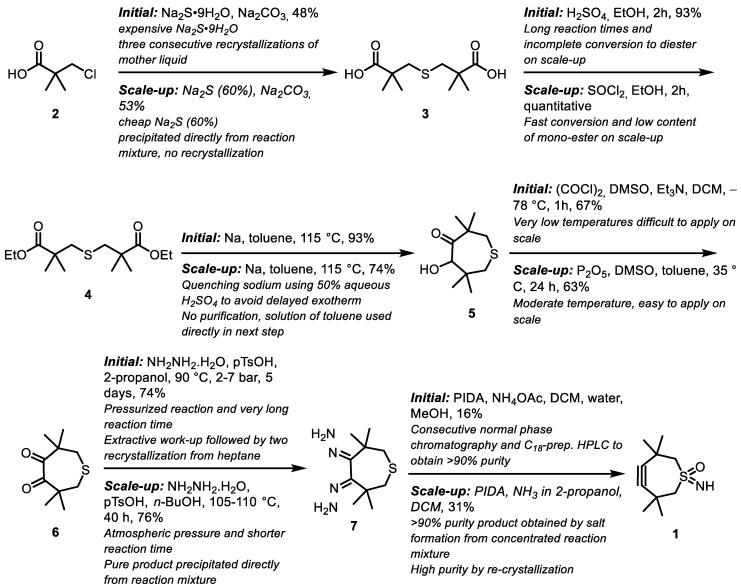
Comparison of initial and scale-up conditions for the preparation of TMTHSI [9] (**1**).

Previous syntheses of TMTHSI have been performed at the gram scale (Scheme 1). For industrial applications, scalability is an important issue to address.

Laboratory-scale synthesis of TMTHSI (**1**) started from commercially available 3-chloro-2,2,-dimethylpropionic acid and has been reported by us [9]. The strategy involved acyloin condensation of diester **4**, followed by Swern oxidation to diketone **6** and one-pot reduction of bis-hydrazone **7** to alkyne with concurrent oxidation of sulfide to sulfoximine. The route had a 3% overall yield and employed consecutive normal and reverse phase chromatographic purification in the last step.

The main shortcomings of the route were defined as a low overall yield of the process, hazardous conditions of the acyloin condensation reaction, cryogenic temperatures employed for the Swern oxidation and scalability difficulties of the chromatographic purification operations.

**Figure 2:**
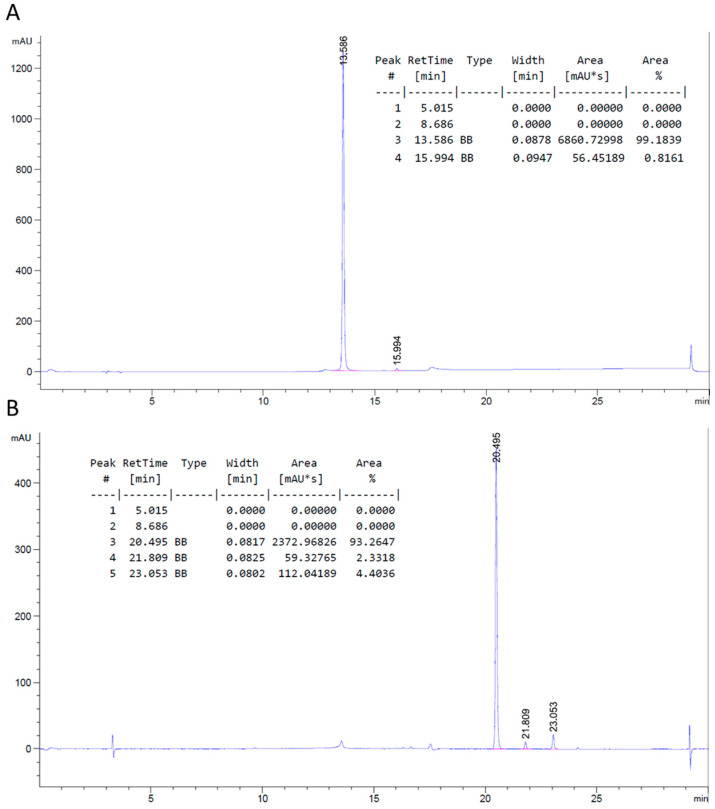
HPLC traces of **4** (**A**) and **7** (**B**) after optimized reaction conditions. Purity based on area percentages inserted. “Peak #” indicates the peak number corresponding to the retention time (“RetTime”). For example: in (**A**) peak number 3 has a retention time of 13.586 min and in (**B**) peak number 3 has a retention time of 20.495 min.

To reach the goal of increasing the synthesis scale by at least a hundred times, reaction and process optimization was undertaken to address these shortcomings. Surprisingly, significant improvement to the deceptively simple thioether **3** formation could not be achieved, though the expensive sodium sulfide nonahydrate could be substituted with 15-times cheaper sodium sulfide with 60% sulfide content. The low yield was caused by the formation of oligomeric sulfide species. Regardless, by washing crude **3** with methyl tert-butyl ether (MTBE) the oligomeric sulfur species were removed and the product could be isolated with 93% HPLC purity after precipitation from water upon acidification. The reaction was performed on an 8 kg scale, producing 3.6 kg of **3** at a 53% yield and 93% HPLC purity. Gram-scale Fischer esterification for the formation of diester **4** was substituted with thionyl chloride activation at a reactor scale to improve the impurity profile, increasing the observed HPLC purity from 86% to 93% (Figure 2A), and yields from 93% to quantitative.

For lab-scale synthesis, sodium was used in the acyloin condensation of **4**. Although employing metallic sodium on a large scale is clearly disadvantageous, no good alternatives of the acyloin reaction were available. Therefore, efforts were focused on ensuring that the reaction could be performed in a safe and reproducible manner. Reaction times were shortened from 20 h to 2–3 h and the reaction was performed in sub-batches to mitigate the risks. During scaling from a 2 L reaction vessel to a 20 L vessel, a decrease in HPLC purity from 93% to 82% was observed. Highly efficient purification could be achieved downstream for compound **7**. The quenched toluene solution gave keto-alcohol **5** at a 67–74% yield and 79–85% HPLC purity, and the obtained toluene solution was used directly in the next step.

Swern oxidation usually requires temperatures below −60 °C [11], which is difficult to achieve upon increasing the scaling. The screening of reaction conditions was therefore undertaken to avoid cryogenic temperatures. Different DMSO-mediated oxidations were tested, but as vicinal diketone and sulfide are prone to overoxidation, only Moffat and P_2_O_5_ modifications produced diketone **6** as the main component. Parikh–Doering modification [12], activations with acetic anhydride or cyanuric chloride produced complex mixtures of components. Diketone **6** was purified via distillation under reduced pressure, and was obtained at a 70% yield in test reactions. Phosphorous pentoxide activation was chosen for scale-up, where a significant amount of increase in a single impurity was observed, which surprisingly did not decrease the yield for obtaining dihydrazone **7**. Solvent screening was performed for dihydrazone 7 formation, and the substitution of 2-propanol by 1-butanol enabled increasing the reaction temperature from 90 °C to 105 °C, which shortened the reaction times from 5–6 days to 40 h and bypassed using pressurized conditions for the reaction. The formed dihydrazone **7** crystallized from the reaction mixture upon cooling and could be obtained at a >98% HPLC purity (Figure 2B), regardless of the observed HPLC purity of diketone **6**. Of **7**, 803 g was obtained starting from 1.3 kg of **5**, at a 48% yield over two steps.

The conversion of compound **7** to TMTHSI (**1**) is a highly delicate reaction, where a number of side-reactions and impurities will form. To decrease the amount of formed impurities, the DCM–MeOH–water ternary water system was changed to a mixture of DCM and 2-propanol, and the ammonia source was changed from ammonium acetate to dissolved ammonia. It was observed that alkyne formation took place readily at <0 °C, but increasing temperature to 10–15 °C was required obtaining the highest content of TMTHSI **1** in the reaction mixture. Chemical yields between 33 and 38% were observed in the crude reaction mixture, which was then filtered to remove ammonium acetate, concentrated and taken up in acetonitrile. Addition of oxalic acid precipitated the formed TMTHSI–oxalate at a good chromatographic purity. The reaction was performed using 90 g of dihydrazone **7**, producing TMTHSI–oxalate at a 23–27% yield.

**Figure 3:**
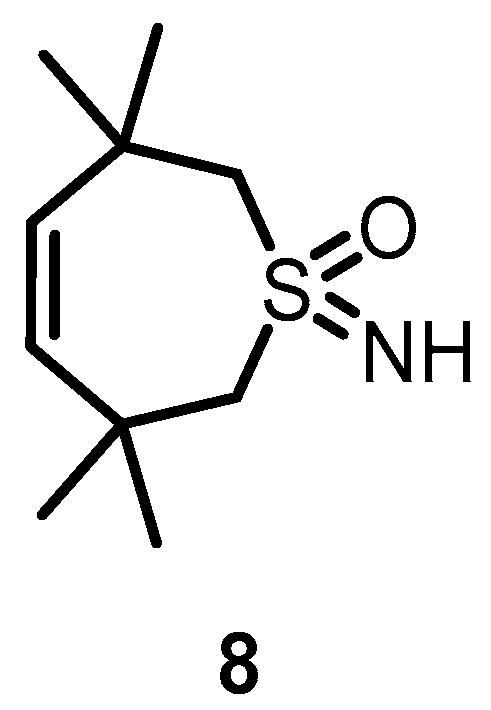
Alkene 8 byproduct of TMTHSI.

After additional investigation via ^1^H NMR, it was observed that even though TMTHSI–oxalate looked highly pure according to HPLC, it contained alkene 8 impurity (Figure 3) at 5–15 mol%. As the alkene did not react in the desired click-reaction, this is a critical impurity, which had to be controlled to low levels (<1%).

**Figure 4:**
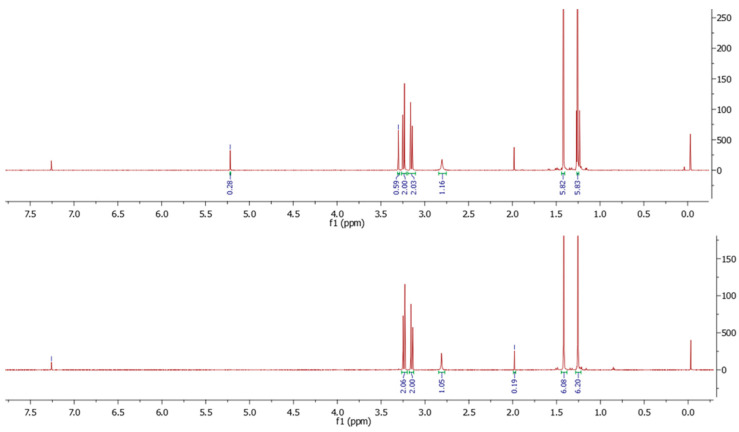
NMR spectra of TMTHSI before (top) and after (bottom) the removal of impurity **8**, as is evident from the disappearance of the alkene signals at 5.22 and 3.30 ppm.

Different approaches starting from chromatographic purification, distillation, and re-forming the salt were tested. Only re-forming the salt via the intermediate free base form of TMTHSI (**1**) had a noticeable purge of alkene impurity, with a moderate yield (60–70%). Different acids were screened to determine if improved purge for alkene impurity could be achieved, but significant improvement could not be achieved. Eventually, it was found that TMTHSI (**1**) as a free base forms crystals in n-hexane and has a similar purge to the salt formation, giving 2–3 fold purge of alkene impurity with an 80% recovery. Using consecutive recrystallizations from n-hexane, <0.5 mol% content of alkene impurity in TMTHSI could be achieved. This result is seen in the NMR spectrum, as depicted in Figure 4. The large scale and high purity which can be obtained indicate that this process is good manufacturing practice (GMP)-compatible. The full synthesis and analysis description of this process can be found in the Supporting Information S1.

**Figure 5:**
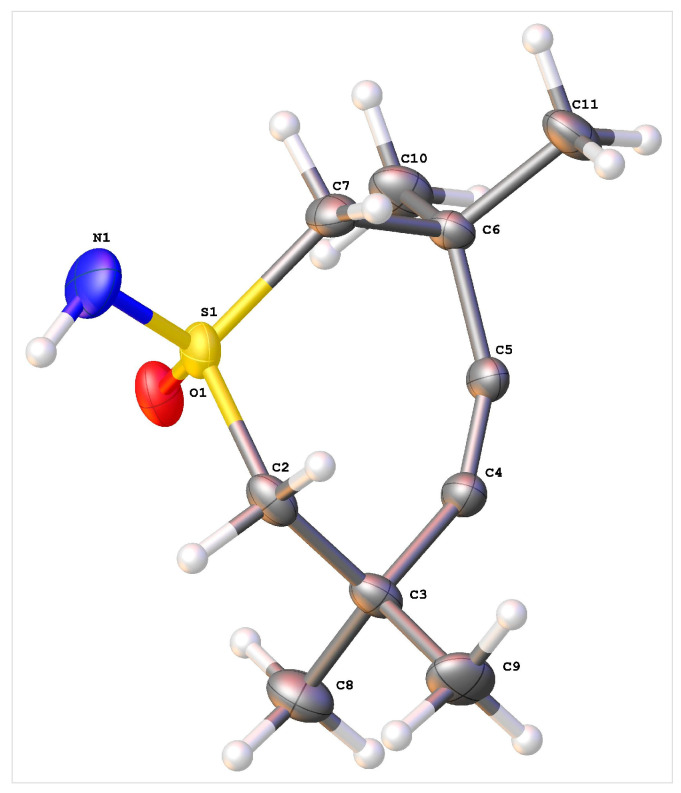
Crystal structure of TMTHSI. Atomic displacement ellipsoids drawn at a 50% probability level.

The obtained crystals were suitable for X-ray crystallographic analysis, which produced the crystal structure, as shown in Figure 5. While this work was in progress, Albada et al. reported the crystal structure of TMTHSI in their paper, denoted as “THS” [13].

This crystal structure is comparable to the findings of Albada et al. [13], with the alkyne bond length at a comparable 1.198 Å, indicating that the crystallization step produced the desired pure compound. The strained alkyne showed a bond angle of 151° to the adjacent single bonds, illustrating the high reactivity of the alkyne.

### 2.2. TMTHSI Stability

**Scheme 2:**
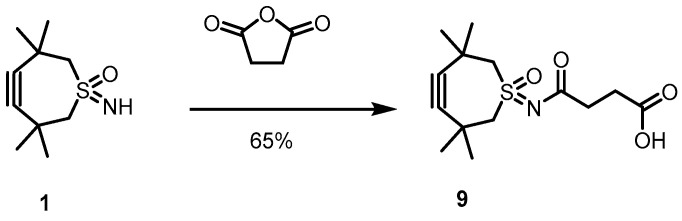
TMTHSI derivatization with succinic anhydride to obtain TMTHSI–succinic acid **9**.

Extensive information on the stability of TMTHSI (-reagents) is required to consider its application in diverse fields. For example, the first step in the use of synthesized TMTHSI **1** will often be derivatization, with a small molecule to introduce a functional handle on the sulfoximine. As previously described [9], succinic anhydride can be used to easily derivatize TMTHSI with a carboxylic acid-reactive moiety, compound **9** (Scheme 2). TMTHSI–succinic acid **9** was obtained at a 66% yield after crystallization (details in Materials and Methods).

**Figure 6:**
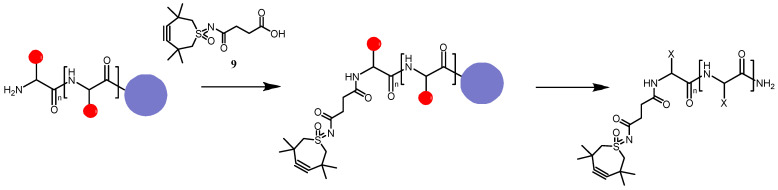
Schematic overview of on-resin introduction of **9** to the *N*-terminus of a peptide, after which the peptide is cleaved from resin (blue) and side groups are deprotected (red).

Investigating its stability is essential because this derivative will often be used for coupling to a click reaction counterpart such as a drug molecule, fluorescent label or targeting ligand. Of special interest is investigation of the stability of TMTHSI during solid-phase peptide synthesis (SPPS), which is performed in organic solvents and requires harsh conditions to cleave and deprotect the peptide. During SPPS, amino acids are sequentially attached to the resin, with side groups protected to prevent undesired side reactions with reactive side groups. This introduces a specificity that can be beneficial for introducing a click linker, as the position can be chosen (Figure 6).

To assess the applicability of TMTHSI in this technique, the stability of derivative **9** was first assessed in commonly used solvents for functionalization, DMF, and acetonitrile at room temperature on a Xevo UPLC–MS (Supporting Information S2). The overlap of UV signals recorded at the start and after 4 h did not show a decrease or shift in the product peak, nor the emergence of new peaks. In combination with the unchanged mass after 4 h, this indicates that TMTHSI–succinic acid **9** is fully stable in an ACN or DMF solution for at least 4 h at room temperature. Moreover, this combination of chromatography and mass detection proved to be a valid method for monitoring the stability of TMTHSI and its derivatives under different conditions.

Next, the use of TMTHSI in SPPS was investigated. A click reagent can be introduced specifically to the *N*-terminus of a peptide after removal of the Fmoc-group (Figure 6). While side-chain groups are protected and the peptide is attached to resin, the functionalization of the *N*-terminus is possible. Specific *N*-terminal modification allows for obtaining peptides of which the biological role of their endopeptidic domains can be investigated.

**Figure 7:**
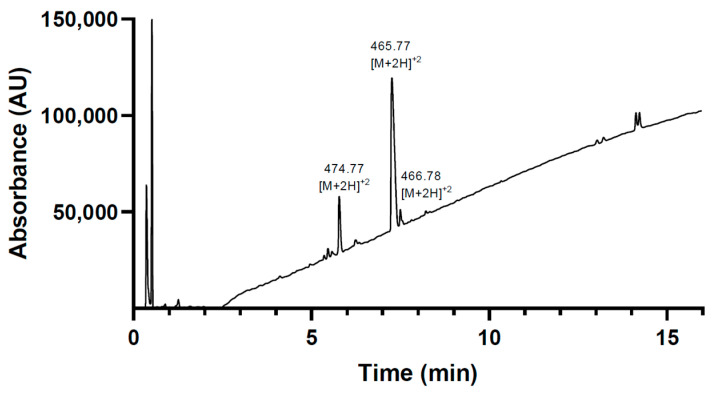
HPLC trace of functionalized LYRAK peptide after cleavage under Fmoc conditions.

A model peptide, LYRAK, was synthesized by Fmoc- and Boc-based SPPS. This resulted in two resin-bound peptides with different side chain-protecting groups, but both with an available *N*-terminus. Both peptides on the resin were functionalized with TMTHSI–succinic acid **9** using HCTU-coupling, and were subsequently deprotected and cleaved. The HPLC traces of functionalized peptide after cleavage and precipitation from diethylether are shown in Figure 7 (Fmoc) and in the Supporting Information Figure S3B (Boc). Use of the Fmoc method for the synthesis and deprotection/cleavage of the peptide using TFA/H_2_O/TIS (90/5/5) showed a large peak at a retention time of 7.3 min, which has a *m*/*z* [M+2H]^+2^ of 465.77, corresponding to the expected mass of 931.16 for the LYRAK–TMTHSI conjugate. Additionally, a smaller peak with retention time 5.7 min corresponded to a *m*/*z* of 474.77, which we assume to be oxidation of the conjugate. In contrast, the Boc method for synthesis and deprotection using HF did not show a product peak with the expected mass.

The stability of TMTHSI under deprotection and cleavage conditions of 90% TFA with 5% TIS and 5% water is unique, as DBCO [14,15] and BCN [16,17] are known to be unstable under these conditions. The need for such a selective on-resin functionalization of the *N*-terminus is illustrated using the LYRAK peptide, since functionalization of this peptide in solution using **9** led to a mixture of peptides with the linker on the *N*-terminus, the linker attached to the lysine side chain, or with two linkers attached to both the *N*-terminus and lysine side chain (Supporting Information Figure S3B).

### 2.3. Antibody Functionalization Using TMTHSI Derivatives

#### 2.3.1. Functionalization of TMTHSI–β-Alanine with FITC

**Scheme 3:**
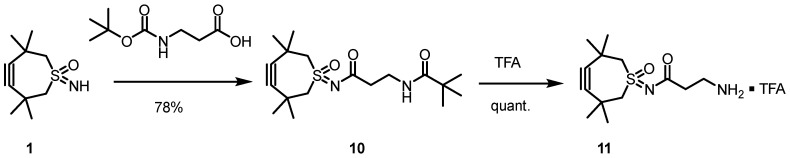
Synthesis of TMTHSI–β-Alanine.

To demonstrate the true bio-orthogonal use of TMTHSI, an antibody was functionalized with a payload. Fluorescein isothiocyanate **12** (FITC) was chosen as a model drug payload to test the application of TMTHSI during the site-specific bioconjugation process. A TMTHSI derivative with an available amine was used for this approach: TMTHSI–β-Alanine **11** (Scheme 3). TMTHSI–β-Alanine can be synthesized from TMTHSI and Boc-protected β-Alanine at a 78% yield as TFA salt.

**Scheme 4:**
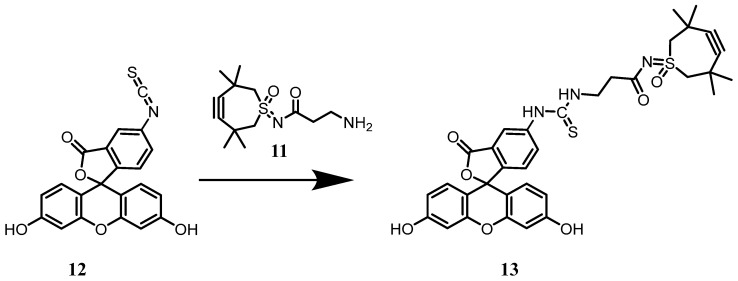
Synthesis of TMTHSI–FITC.

The isothiocyanate derivative could be readily coupled via the amine of the TMTHSI–β-Alanine derivative **11** to produce clickable fluoresceine **13** (TMTHSI–FITC) (Scheme 4).

TMTHSI–FITC **13** was obtained at a 30% yield after purification using reverse-phase chromatography, and was characterized by ^1^H-NMR and LC–MS (See Section 4: Materials and Methods).

#### 2.3.2. Site-Selective Antibody Functionalization with Azide Moieties

**Figure 8:**
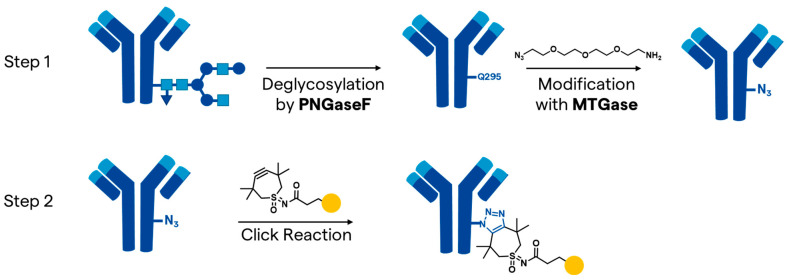
Schematic illustration of the two-step conjugation approach. Step 1: The native glycans on the antibody constant region are trimmed enzymatically by the enzyme PNGaseF to make an adjacent glutamine residue (Q295) available for modification with an azide–amine linker molecule by a transglutaminase (MTGase). Step 2: The antibody–azide intermediate is clicked to a TMTHSI payload molecule to produce a stable and homogeneous DAR2 antibody conjugate. For clarity, only one glycan and introduced azide on one heavy chain are shown.

**Figure 9:**
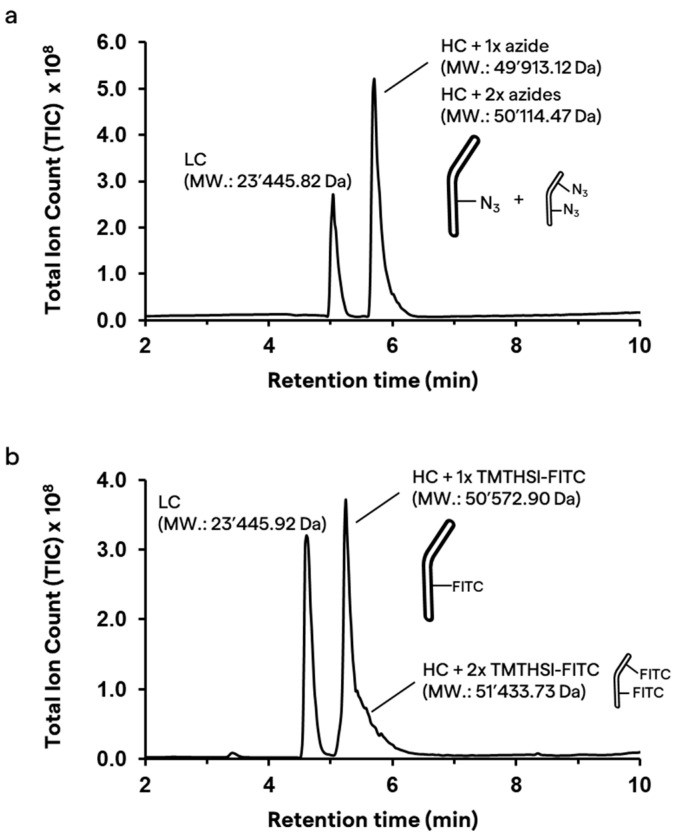
LC–MS trace of azide-modified antibody intermediate and antibody–FITC conjugate. (**a**) Analysis after enzymatic modification of the antibody with the azido linker. Only the heavy chain (HC) was modified, the main species being HC with one azide molecule. Smaller amounts of HC carrying two azides were also detected. (**b**) Analysis of the conjugated product, resulting from the reaction with 5.0 eq. TMTHSI–FITC after 4 h. The main conjugated species is HC carrying one TMTHSI–FITC molecule. Double-modified HC eluted slightly later due to increased hydrophobic interactions.

Bevacizumab, a well characterized IgG1 antibody, was chosen for testing the conjugation of TMTHSI–fluorescein **13** following a site-selective conjugation approach targeting the conserved glutamine Q295. A preceding deglycosylation step using PNGaseF was necessary to make the Gln residue available for modification. For the introduction of the TMTHSI reaction partner, the antibody was modified with an azide handle following a procedure described in the literature that uses a microbial transglutaminase (MTGase) for the site-specific modification of Q295 in the Fc portion of the antibody with an azide-containing amine linker molecule [18] (Figure 8, step 1). LC–MS analysis was used to analyze the modified antibody. Under reducing conditions using DTT treatment during sample preparation, the light chain (LC) and heavy chain (HC) of the antibody can be separated, enabling the detection of modified species via the MS read-out. Analysis via LC–MS confirmed the site-specific modification of the heavy chain (HC) with the azide molecule (Figure 9a). HC carrying one azide linker molecule (mass detected: 49,913.12 Da; Δm to H0 = 201.2 Da, which corresponds to the azide handle) was the main species, with no unmodified HC (H0) detectable. The mass spectrum also showed the presence of HC modified with two azide molecules, which likely results from an unspecific modification of another Gln residue by MTGase [19,20]. No modification of the light chain was detected.

### 2.4. Click Reaction with TMTHSI–FITC

**Figure 10:**
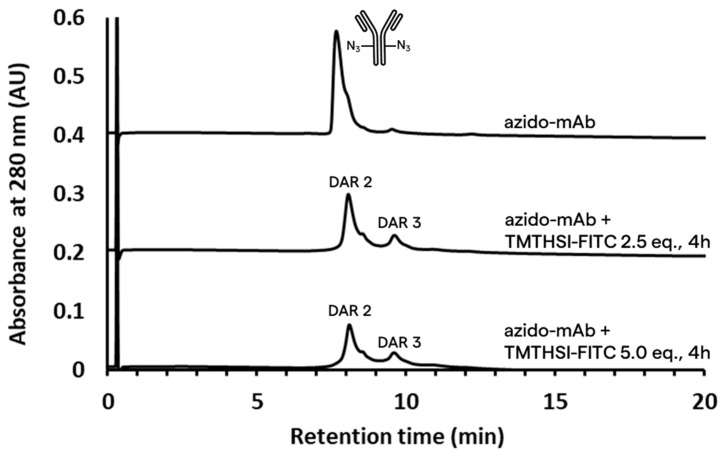
HIC trace of antibody–fluorescein conjugate after 4 h with 2.5 eq. (**middle**) and 5.0 eq. (**bottom**) TMTHSI–FITC in comparison to the antibody–azide intermediate (**top**). Due to the hydrophilic nature of both TMTHSI and the fluorescein payload, the retention time of the DAR2 species is only slightly shifted compared to the azido-mAb, whereas the shift is more pronounced for the DAR3 species. Both reactions gave quantitative conversion to conjugated product, with an average DAR of ca. 2.3.

The performance of TMTHSI in a conjugation reaction with azide-modified bevacizumab was tested at 2.5 and 5.0 eq. excess per mol antibody, which corresponded to approx. 1.25 and 2.5 eq. per available azide (Figure 8, step 2). The reaction was monitored via LC–MS after 1 h, 2 h, and 4 h. Additionally, the final product was further characterized by HIC and SEC. The MS data confirmed the successful conjugation of the TMTHSI model payload to the azide-modified antibody (Figure 9b). The heavy chain modified with one molecule, TMTHSI–FITC, was the main species. As was expected from the azide modification step, we also detected smaller amounts of the species that corresponded to a HC, plus two TMTHSI–FITC payloads. The average (model) drug–antibody ratio (DAR) was, thus, determined to be 2.3 (determined by HIC) for both the reaction with 2.5 and 5 eq. of TMTHSI–FITC per mAb (Figure 10), slightly higher than the theoretical DAR of 2.0 based on the underlying conjugation strategy. No significant changes in DAR were observed via LC–MS during the course of 4 h, and the click reactions were essentially complete after only 1 h. Importantly, the reaction with 2.5 eq. TMTHSI–FITC, which corresponds to only ca 1.25× excess of click reagent over available azide, showed an equally good performance as the reaction with 5 eq. The relatively low amount of (often expensive) payload required for the full conversion is attractive as it is quite cost-effective.

The fluorescein-conjugated antibodies were also analyzed via size-exclusion chromatography (SEC). Comparison with the azide-modified bevacizumab showed that high molecular-weight species (HMWS) increased by ca. 6–8% upon the clicking of the fluorescein payload (Supporting Information S5). The increase in HMWS can be mostly attributed to the known propensity of the chosen antibody for aggregation upon buffer changes and concentration steps [21,22].

## 3. Conclusions

TMTHSI is an exceptionally promising new class of click reagents. Scale-up up to 100 g is demonstrated, yielding a high purity during a GMP-compatible process. TMTHSI derivatives can be applied in the development of click-modified bioconjugates and clinical evaluations. The stability of TMTHSI is unique, as shown through compatibility with the acidic TFA cleavage cocktail used in Fmoc-based SPPS. This stability opens up the possibility of an on-resin modification of peptides, specifically at the N-terminus when using Fmoc protection chemistry. Along with peptides, TMTHSI can also be used for the functionalization of antibodies. The model system for the conjugation of a small molecule payload to an antibody using a TMTHSI-based probe confirmed the suitability of TMTHSI in a bioconjugation setting. The reactivity of the seven-membered sulfoximine-based strained alkyne allows for very short reaction times and lower amounts of equivalents of the payload, which is highly beneficial for a cost-effective process, and hence a broad applicability in the biomedical field is foreseen.

## 4. Materials and Methods

### 4.1. General

Commercially sourced reagents and solvents were used without any further purification.

Nuclear magnetic resonance (NMR) spectra were recorded on a Bruker Avance-III 700 MHz spectrometer at 25 °C. Chemical shifts (δ) are reported in parts per million (ppm), and were referenced relative to the solvent residual signal (^1^H—7.26 ppm for CDCl_3_; ^13^C—77.16 ppm for CDCl_3_). The coupling constants (J) are reported in hertz (Hz). High-performance liquid chromatography (HPLC) analyses were performed on an Agilent 1100 and 1200 series machines equipped with a diode array detector (DAD). A reverse-phase column (XSelect CSH C18, 5 µm, 4.6 mm × 250 mm) was used using water (0.1% phosphoric acid) and acetonitrile (0.1% phosphoric acid) from 5% organic to 95% organic components, with detection at 210 nm. Mass spectra were recorded on an API 3200 QTRAP machine using electrospray ionization (ESI).

Peptide and stability measurements were performed on a Waters XEVO QTOF G2 mass spectrometer. The system was equipped with an Acquity H-class solvent manager, an FTN sample manager and a TUV detector. The column used was a reverse-phase C18 column (Waters, Acquity PST 130A, 1.7 μm 2.1 × 50 mm i.d.), and the column temperature was held at 40 °C. Buffer A consisted of 0.1% formic acid in water, and buffer B consisted of 90% ACN, 9.9% H_2_O and 0.1% formic acid.

Liquid chromatography mass spectrometry (LC–MS) for analysis of the bioconjugates was recorded on a machine from Thermo Scientific. A reverse-phase column (BioResolve) was used with water and acetonitrile (both containing TFA as the acidic modifier) from 25% organic to 90% organic component, with detection at 280 nm.

Hydrophobic interaction chromatography (HIC) analyses were recorded on an HPLC device from Agilent, using a HIC column (Tosoh) with a butyl-based adsorbent. Mobile phase A was a sodium phosphate buffer (pH 7.0) containing ammoniumsulfate. Mobile phase B was a phosphate buffer with isopropanol as an organic co-solvent. Elution was carried out by increasing mobile phase B from 0 to 100%, with detection at 280 nm.

Size-exclusion chromatography (SEC) analyses were recorded on a HPLC device from Waters, using a gel filtration column from Waters. An isocratic elution was carried out using a PBS buffer (pH 7.4) containing isopropanol, with detection at 280 nm.

### 4.2. X-Ray Crystallography

Data collection: APEX3 Ver. 2016.9-0 (Bruker-AXS, Karlsruhe, Germany, 2016); cell refinement: SAINT V8.40B (2016); data reduction: SAINT V8.40B (2016); program(s) used to solve structure: SHELXT 2018/2 (Sheldrick, 2018 [23]); program(s) used to refine structure: SHELXL 2018/3 (Sheldrick, 2015 [24]); molecular graphics: Olex2 1.5 [25]; software used to prepare material for publication: Olex2 1.5 [25]. Crystal data and additional details can be found in Supporting Information S6.

### 4.3. Synthesis of ***9***

TMTHSI hydrochloride salt (15 g, 63.6 mmol, 1 eq) was placed in a 500 mL round-bottomed flask. Next, 300 mL dichloromethane was added, followed by a slow addition of N,N-diisopropylethylamine (28.8 g, 223 mmol, 3.5 eq.) in the course of 15 min. Succinic anhydride (9.6 g, 95.4 mmol, 1.5 eq.) was added portion-wise and the reaction mixture was stirred at an ambient temperature for 3 h. Thereafter, 400 mL of 10% aqueous KHSO_4_ solution was added, and the organic layer was separated and dried over anhydrous Na_2_SO_4_. The salt was filtered and volatiles were removed under reduced pressure at a 35 °C water bath. The residue was dried at 0.5 torr for 20 min, affording 20 g of grey solid. The crude product was recrystallized from 110 mL of acetonitrile. The filter cake was washed with diethyl ether and dried under reduced pressure at room temperature. This afforded 12.5 g of compound **9** as cream crystals at a 66% yield and 99.1% HPLC purity (210 nm). ^1^H NMR (700 MHz, CDCl_3_) δ 3.70–3.59 (m, 4H), 2.72–2.58 (m, 4H), 1.51 (s, 6H), 1.26 (d, J = 17.5 Hz, 6H). ^13^C NMR (176 MHz, CDCl_3_) δ 180.33, 177.94, 101.52, 68.05, 34.28, 34.10, 29.92, 27.84, 27.05. MS–ESI calculated for C_14_H_22_NO_4_S (M+H^+^)^+^ 300.12, found 300.24. The NMR spectrum of **9** is depicted in Supporting Information S7.

### 4.4. Peptide Coupling with ***9*** and Cleavage

A quantity of 5 µmol on-resin LYRAK was used, with 5× excess TMTHSI–succinic acid (25 µmol). The 5× excess was used here, taking into consideration that this is a heterogeneous reaction system which is less efficient than a homogeneous resin system. A 0.9:1 ratio of HCTU to TMTHSI–succinic acid, and a 2.3:1 ratio of DiPEA to HCTU (900 μL 0.005 M HCTU in DMF and 3.6 μL 2× diluted DiPEA) was added to 25 µmol TMTHSI–succinic acid. After a 3 min activation, this was added to Fmoc and Boc on-resin LYRAK. After 20 min, the reaction was stopped by wash steps with DMF and DCM. The product was cleaved using the standard cleavage protocols:

Boc: Peptides synthesized using Boc chemistry were cleaved from resins via HF cleavage. The on-resin peptides were cleaved for 1 h in HF, with the addition of 4% p-cresol (scavenger). The reaction mixture was suspended in diethylether, which was subsequently filtered. The peptide was dissolved in 55:45 H_2_O:ACN with 0.05% FA, and analyzed on UPLC–ESI–MS.

Fmoc: Peptides synthesized with Fmoc chemistry were cleaved from the resins via TFA treatment. The peptidyl-resins were cleaved for 3 h with a solution consisting of 90% TFA, 5% H_2_O, and 5% triisopropylsilane (TIS). After this, the solution was pressed through a filter to remove the resin. Next, TFA was evaporated and diethylether was used to precipitate the peptide. After centrifugation for 3 min at 5000 rpm until a pellet was formed, the ether was removed from the precipitate. Subsequently, the peptide was dissolved in 55:45 H_2_O:ACN with 0.05% FA, and analyzed on UPLC–ESI–MS.

### 4.5. Synthesis of ***11***

Boc-protected β-alanine (4.5 g, 24 mmol, 1 eq.), HOBt·H2O (4.4 g, 29 mmol, 1.2 eq.), EDC (5.5 g, 29 mmol, 1.2 eq.), and 84 mL of dichloromethane were placed into a 250 mL round-bottomed flask equipped with a magnetic stirring bar. The mixture was stirred for 25 min at an ambient temperature under inert gas. TMTHSI hydrochloride salt (6.7 g, 29 mmol, 1.2 eq.) in 60 mL of dichloromethane was mixed with triethylamine (4.0 mL, 29 mmol, 1.2 eq.) and a white suspension was obtained. The suspension was added to the reaction mixture and stirring was continued at ambient conditions under inert gas for 24 h. The reaction mixture was washed with 330 mL of 6% aqueous sodium bicarbonate solution, followed by 190 mL of 5% aqueous KHSO_4_ solution and 80 mL of brine. The separated organic layer was dried over anhydrous sodium sulfate, filtered and concentrated under reduced pressure. The crude product was purified using normal-phase column chromatography eluting with n-heptane: the MTBE gradient elution from 0% to 80% MTBE. The obtained product comprised 6.9 g of white solid at a 78% yield.

Boc-protected TMTHSI conjugate with β-alanine obtained in the previous step (0.93 g, 2.5 mmol, 1 eq.) was dissolved in 3.8 mL of trifluoroacetic acid (TFA) and stirred for 20 min. The reaction mixture was poured into 20 mL of diethyl ether and seeded with a small amount of product. After 15 min, precipitate formed, and was filtered and washed with diethyl ether. Of TMTHSI-β-alanine conjugate **11**, 0.68 g was obtained as a TFA salt at a quantitative yield and 100% HPLC purity (210 nm). ^1^H NMR (700 MHz, CDCl_3_) δ 7.88 (s, 3H), 3.83 (d, J = 14.2 Hz, 2H), 3.52 (t, J = 9.1 Hz, 2H), 3.24 (d, J = 5.1 Hz, 2H), 2.76–2.65 (m, 2H), 1.50 (s, 6H), 1.26 (s, 6H). MS–ESI calculated for C_13_H_23_N_2_O_2_S [M+H^+^]^+^ 271.14, found 271.1. The NMR spectrum of **11** is depicted in Supporting Information S7.

### 4.6. Synthesis of ***12***

The TFA salt of TMTHSI–β-Alanine (100 mg, 0.271 mmol) was dissolved in DCM (12 mL) and DIPEA (6 eq., 1.624 mmol) was added. The resulting mixture was stirred at the RT. After 5 min, 5′ isothiocyanate (1.2 eq., 0.325 mmol) was added portion-wise. The mixture was stirred at the RT for 10 min. The solvent was evaporated and the residue was loaded on a C18aq. cartridge (2 × 50 g) eluted with water/ACN from 95/5 to 0/1 in a gradient. Relevant fractions from each injection were collected and acetonitrile was evaporated (bath at 20° C). The aqueous solutions were dried over a lyophilizer to afford the product as 1/1 (mol/mol) mixture with DIPEA. This material was thus redissolved in a minimal amount of acetonitrile/water (1/1), loaded on an SCX cartridge (1 g) and recovered by washing the SCX with acetonitrile/water (2/1). Acetonitrile was evaporated (20 °C) and the resulting water solution was lyophilized to afford the clean product as an orange solid (yield 30%).

1H NMR (400 MHz, DMF) δ = 10.33 (s, 2H), 10.21 (br s, 1H), 8.50 (s, 1H), 8.15 (br t, J = 5.5 Hz, 1H), 7.85 (br d, J = 8.0 Hz, 1H), 7.23 (d, J = 8.3 Hz, 1H), 6.70 (d, J = 2.3 Hz, 2H), 6.67–6.59 (m, 4H), 4.01 (d, J = 14.0 Hz, 2H), 3.88–3.81 (m, 2H), 3.78 (d, J = 14.0 Hz, 2H), 2.65–2.58 (m, 2H), 1.40 (s, 6H), 1.21 (s, 6H); *m*/*z* [M+H^+^]^+^ 660; UV purity (254 nm) 94%.

### 4.7. Antibody Modification and Conjugation

For the glycosylation step, a buffer change was performed by desalting columns to change the buffer for bevacizumab (starting amount of protein: ca. 170 mg) from its formulation buffer to 1× PBS (pH 7.4). The protein concentration was determined by a SoloVPE measurement (ext. coeff. λ = 280 nm: 1.70 mL/(mg×cm)). An appropriate amount of the deglycosylation enzyme PNGaseF (6 U/mg of antibody) dissolved in water was added to the antibody solution (19 mg/mL). The mixture was incubated at 37 °C overnight while slightly stirring. The deglycosylated mAb was purified via Protein A chromatography, eluted in 0.1 M glycine (pH 2.8), followed by buffer change to 1× PBS.

To initiate the enzymatic modification reaction, the deglycosylated mAb (8.4 mg/mL) was mixed with 80 eq of azido-PEG3-amine (solution diluted in water), followed by the addition of an appropriate amount of MTGase (6 U MTGase/mg mAb). The mixture was incubated overnight at 37 °C while slightly stirring. The azide-modified mAb was purified via Protein A chromatography, eluted in 0.1 M glycine (pH 2.8), followed by buffer change to 1× PBS. A sample was pulled for analysis of the modification reaction via LC–MS. The rest of the solution was stored at −80 °C until further use for the click reactions.

### 4.8. Click Reaction of TMTHSI–FITC

Bevacizumab modified with the azide linker was thawed at room temperature and diluted with 1× PBS buffer to a concentration of 6.0 mg/mL. Of this solution, 2× 3 mL was transferred into two separate reaction containers. DMSO was added to reach a final concentration of 10% (*v*/*v*), directly followed by the addition of TMTHSI–FITC (10 mM in DMSO) at 5.0 and 2.5 eq. with respect to the mAb–azide intermediate. The conjugation reactions were incubated at room temperature, while slightly stirring. In order to monitor the progress of the click reaction, the reactions were quenched after 1 h, 2 h, and 4 h by the addition of an excess of azido-PEG3-amine to remove remaining TMTHSI–FITC. The quenched solutions were purified via desalting columns and subjected to analysis via LC–MS (the 4 h timepoints were additionally analyzed via HIC and SEC).

## Data Availability

Data is contained within the article and supplementary material.

## Supplementary Materials

The following supporting information can be downloaded at: https://www.mdpi.com/article/10.3390/ph16081155/s1, S1: Large scale production of TMTHSI; S2: stability of TMTHSI-succinic acid in DMF and ACN; S3: TMTHSI-succinic acid coupling to LYRAK peptide and HF cleavage; S4: Conjugation of TMTHSI-succinic acid to LYRAK in solution; S5: SEC trace of azide-modified antibody; S6: Structure report for TMTHSI; S7: NMR spectra of TMTHSI succinic acid 9 and TMTHSI-β-alanine 11.
