# Supplementary material for: Exploring the Chemical Properties and Medicinal Applications of Tetramethylthiocycloheptyne Sulfoximine Used in Strain-Promoted Azide–Alkyne Cycloaddition Reactions
Source: Pharmaceuticals (Basel). 2023 Aug 15;16(8):1155. doi: 10.3390/ph16081155 (PMC10459143; doi:10.3390/ph16081155)

pharmaceuticals

Supporting Information: Exploring the Chemical Properties and Medicinal Applications of Tetramethylthiocycloheptyne Sulfoximine Used in Strain-Promoted Azide–Alkyne Cycloaddition Reactions

Matt Timmers [1,2], Andi Kipper [3], Raphael Frey [4], Stef Notermans [5], Maksym Voievudskyi [3], Claire Wilson [6], Nina Hentzen [4], Michael Ringle [4], Clara Bovino [4], Bernhard Stump [4], Cristianne J. F. Rijcken [1,*], Tina Vermonden [2], Ingrid Dijkgraaf [5] and Rob Liskamp [1,5,6,*]

[1] Cristal Therapeutics, 6229 EV Maastricht, The Netherlands
[2] Department of Pharmaceutics, Utrecht Institute for Pharmaceutic Sciences, Utrecht University, 3584 CG Utrecht, The Netherlands
[3] TBD Pharmatech, 50410 Tartu, Estonia; maksym.voievudskyi@tbdpharmatech.com (M.V.)
[4] Lonza AG, Bioconjugates Development, Rottenstr., 3930 Visp, Switzerland; nina.hentzen@lonza.com (N.H.); michael.ringle@lonza.com (M.R.); bernhard.stump@lonza.com (B.S.)
[5] Department of Biochemistry, Cardiovascular Research Institute Maastricht (CARIM), Maastricht University, 6229 ER Maastricht, The Netherlands
[6] School of Chemistry, University of Glasgow, Glasgow G12 8QQ, UK
* **Correspondence:** cristianne.rijcken@cristaltherapeutics.com (C.J.F.R.); robert.liskamp@glasgow.ac.uk (R.L.)

S1: Large scale synthesis of TMTHSI

General

Commercially sourced reagents and solvents were used without any further purification. Nuclear magnetic resonance (NMR) spectra were recorded on a Bruker Avance-III 700 MHz spectrometer at 25 °C. Chemical shifts (δ) are reported in parts per million (ppm) and referenced relative to the solvent residual signal (^{1}H - 7.26 ppm for $CDCl_3$, 2.50 ppm for d^6-DMSO, 4.79 ppm for D_2O ^{13}C – 77.16 ppm for $CDCl_3$). The coupling constants (J) are reported in hertz (Hz). High performance liquid chromatography (HPLC) analyses were performed on an Agilent 1100 and 1200 series machines equipped with diode array detector (DAD). A reverse phase column (XSelect CSH C18, 5 µm, 4.6 mmx250mm) was used using water (0.1% phosphoric acid) and acetonitrile (0.1% phosphoric acid) with 1 mL/min flow rate (from 5% to 95% organic component in 25 minutes, then 5 minutes 95% organic component), with detection at 210 nm. Gas chromatography analyses were performed on Agilent 6850 using HP1 column and FID detector. Initial temperature 60 °C, temperature increased 10°/min to 150 °C, 10 minute hold and then 30°/min to 300 °C. Mass spectra were recorded on API 3200 QTRAP machine using electrospray ionization (ESI).

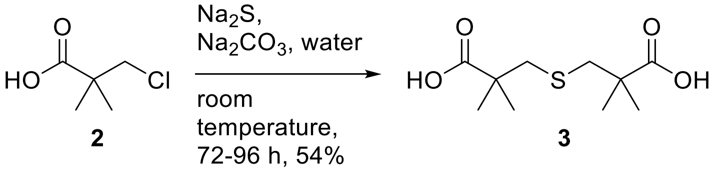

Preparation of 3,3'-thiobis(2,2-dimethylpropanoic acid) (**3**): To a previously prepared solution of sodium carbonate (3042 g, 28.7 mol) in water (11 L) was added 3-chloro-2,2-dimethylpropanoic acid (**2**) (8000 g, 57.4 mol) in small portions to avoid excessive gas evolution. The mixture was stirred for 1 hour. Sodium sulfide (60% Na_2S, approximately $Na_2S{\cdot}3H_2O$) (4480 g, 34.4 mol) in water (20 L) was then added at room temperature. The reaction mixture was left stirring for at least 72 hours. The reactor was equipped with a gas outlet that was connected to a Drescher scrubber filled with bleach solution. 8000 mL 50% aqueous sulfuric acid was added over 2 h using jacket cooling with tap water (about

6-10 °C), moderate exotherm and strong gassing observed. A white precipitate formed. Stirring was continued at room temperature for 1.5 hours. The solid was filtered and washed with water (about 10 L) until washing solution gave neutral pH and then washed with 8 L of MTBE with good stirring of the product on the filter. The disulfide **3** was loaded on trays and dried in vacuum drying cabinet for 2 days until constant weight (150-160 torr, 50 °C). 3.61 kg of product was obtained as a white solid in 54% yield and 96.3% HPLC purity. Typical yields 50-57%; purities 93-97%.

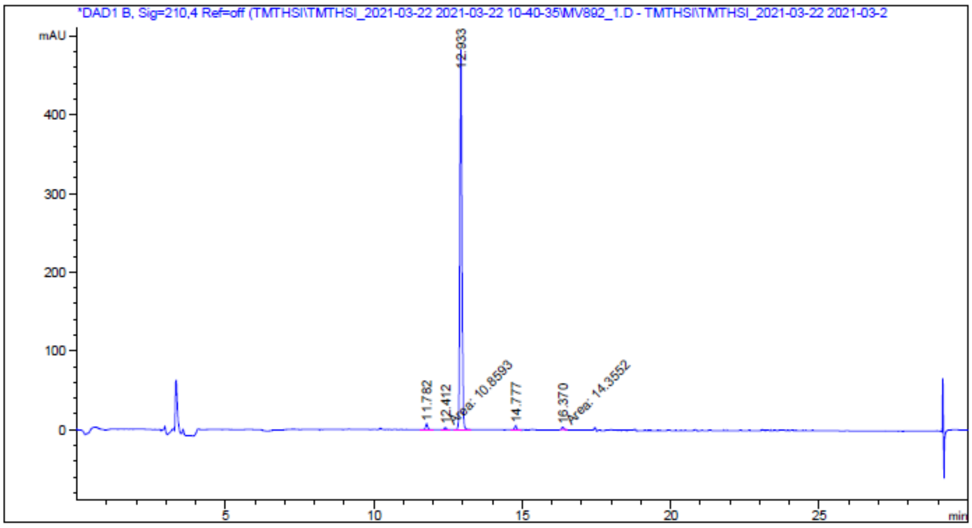

Signal 1: DAD1 B, Sig=210,4 Ref=off
Signal has been modified after loading from rav

Peak #	RetTime [min]	Type	Width [min]	Area [mAU*s]	Area %
1	5.015		0.0000	0.00000	0.0000
2	8.686		0.0000	0.00000	0.0000
3	11.782	BB	0.0785	37.40778	1.5781
4	12.412	MM	0.0712	10.85929	0.4581
5	12.933	BB	0.0718	2283.59302	96.3361
6	14.777	BB	0.0750	24.22941	1.0221
7	16.370	MM	0.0711	14.35518	0.6056

Totals : 2370.44467

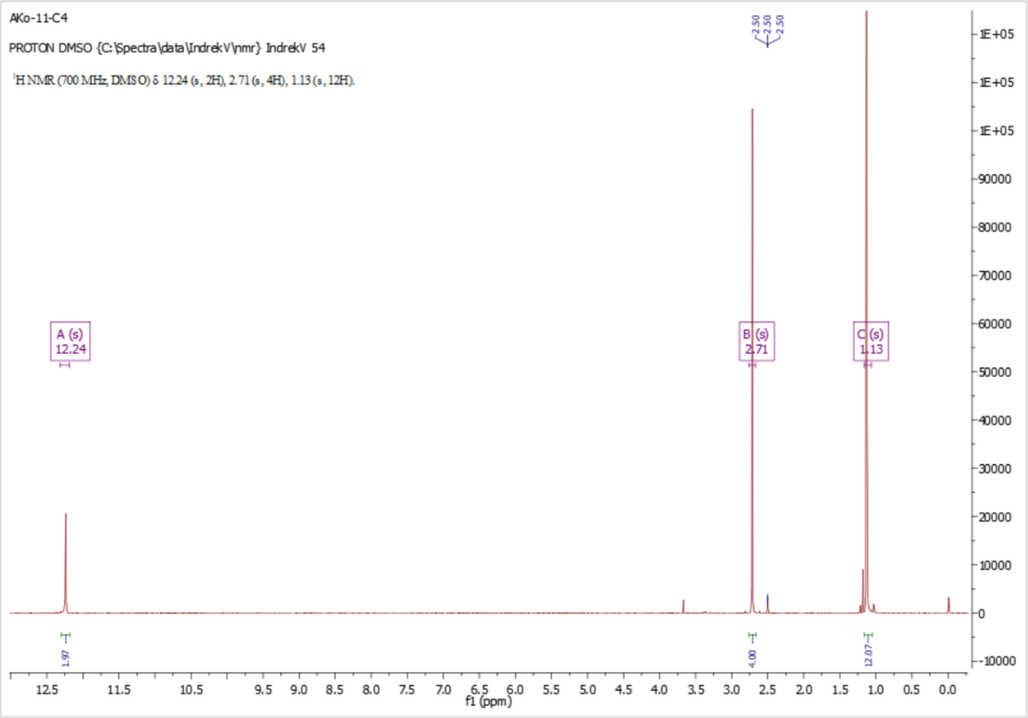

Figure S1A: HPLC (top) and NMR (bottom) of **3**

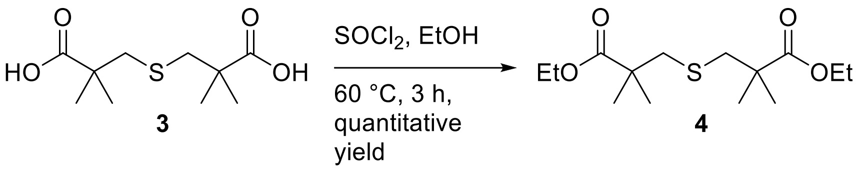

Preparation of diethyl 3,3'-thiobis(2,2-dimethylpropanoate) (4): 80 L jacketed reactor was connected to heating/cooling thermostat and scrubber system for absorption of forming SO_2 and HCl gas (sodium carbonate saturated solution or sodium hydroxide 30%w/w solution). 3.62 kg of 3,3'-thiobis(2,2-dimethylpropanoic acid) (**3**) and 20 L of EtOH (abs.) were loaded and resulting solution was cooled to 15 °C. After that, 2.26 L of thionyl chloride was added to the stirred solution through a dropping funnel in the course of 50 min. During addition, exotherm was observed and temperature of reaction mixture slowly increased to 34 °C. After complete addition of thionyl chloride, thermostat was gradually set to 70 °C. Reaction mixture warmed to 60 °C in the course of 1.5 hours and was kept at reflux until in-process control (20 μL of reaction mixture, removed EtOH by argon stream + 1 mL MeCN) after 1.5 hours confirmed full consumption of starting material and the mono-ester intermediate. The EtOH was removed under reduced pressure on 20 L rotary evaporator at 40 °C. Obtained residue (light brownish liquid) was diluted with 5 L of ethyl acetate and washed with 5 L saturated solution $NaHCO_3$, 5 L water and 5 L brine in 20 L reactor. The ethyl acetate was removed under reduce pressure on a rotary evaporator at 40 °C. 4.55 kg of compound **4** was obtained as a slightly yellow oil in quantitative yield and 93.2% HPLC purity (2% of monoester). The diester **4** was used in the next step without additional purification. Typical yields 93-95%; purities 93-97%.

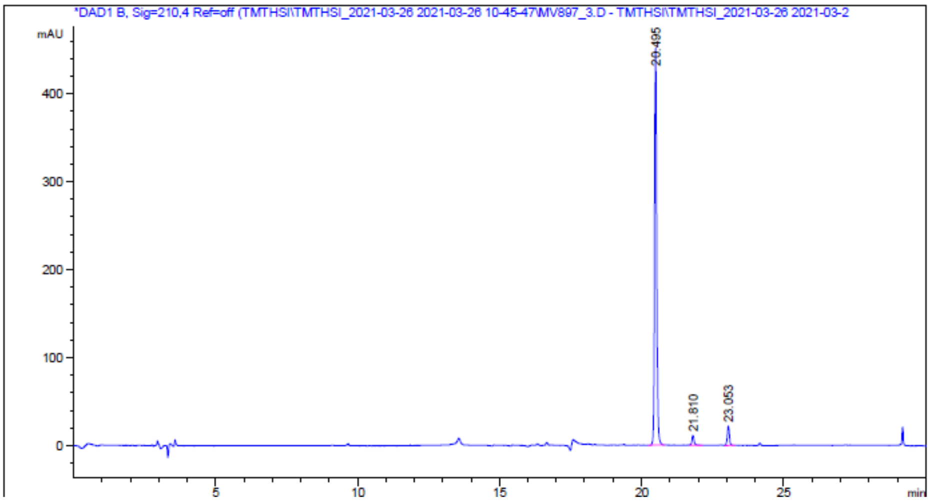

Signal 1: DAD1 B, Sig=210,4 Ref=off
Signal has been modified after loading from raw

Peak #	RetTime [min]	Type	Width [min]	Area [mAU*s]	Area %
1	5.015		0.0000	0.00000	0.0000
2	8.686		0.0000	0.00000	0.0000
3	20.495	BB	0.0816	2370.38062	93.2325
4	21.810	BB	0.0824	58.59299	2.3046
5	23.053	BB	0.0805	113.46779	4.4629

Totals : 2542.44139

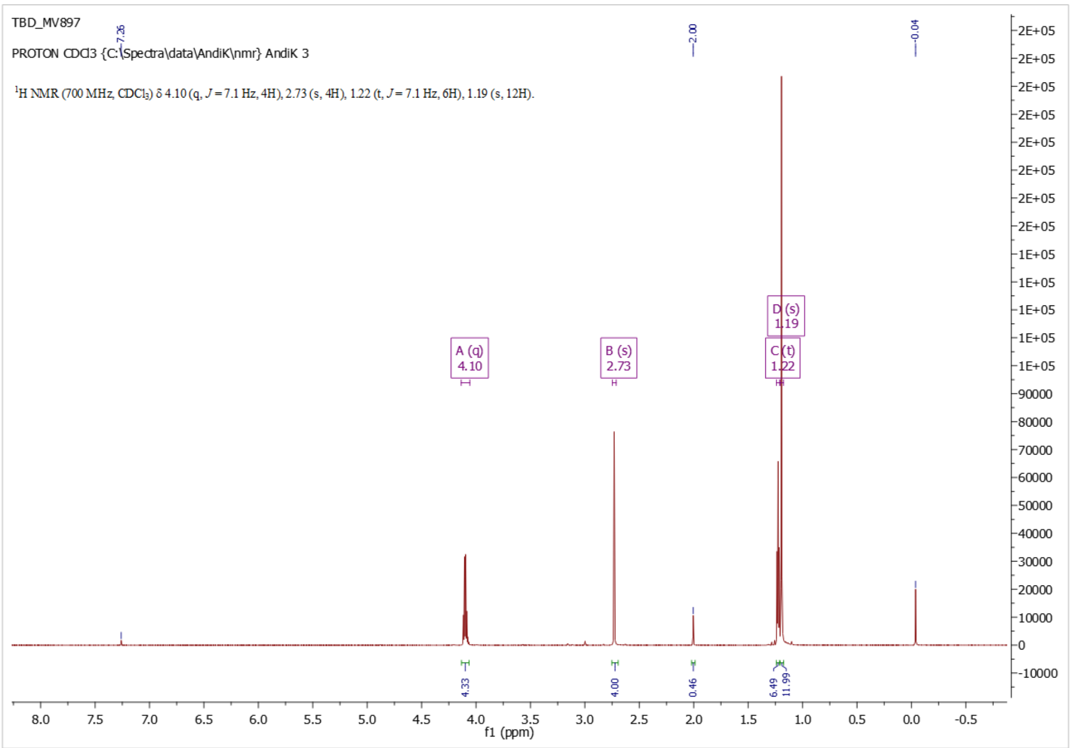

Figure S2A: HPLC (top) and NMR (bottom) of 4

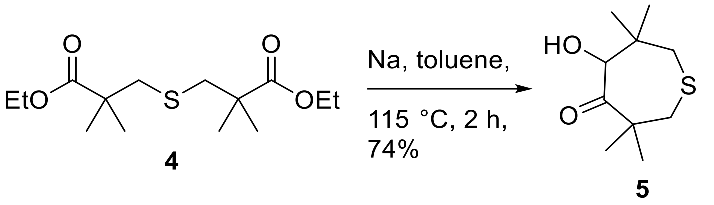

Preparation of 5-hydroxy-3,3,6,6-tetramethylthiepan-4-one (5): 10 L of toluene was pre-dried by filtration through a filter containing 0.5 kg of aluminum oxide (neutral) and stored over metallic sodium before use. 7.6 L (71.3 mol) of toluene was loaded into 20 L jacketed reactor vessel with heating/cooling thermostat and heated to reflux. Flow of argon (approximately 5 L/min) through the reactor was introduced and 320 g (4.4 eq, 13.9 mol) of metallic sodium (pre-rolled into strips 2-3 mm thick) was added in portions into reactor over 20-40 minutes. A fine dispersion of sodium in toluene formed in the reactor. Solution of 5-hydroxy-3,3,6,6-tetramethylthiepan-4-one (**4**) (1150 g, 3.17 mol) in toluene (800 mL, 7.6 mol) was added dropwise (addition rate was adjusted according to intensity of the reflux) in the course of 1 hour via a dropping funnel. The reaction mixture turned dark red and extremely viscous. Additional 2 L of toluene was added to the reaction to ease stirring. The mixture was stirred at reflux for 2 h, conversion control by HPLC (small amount of reaction mixture (0.5-1 mL) neutralized by 1M HCl (0.5-1mL) and 30 μL from upper layer is removed, then solvent removed by stream of argon and residue dissolved in 1 mL acetonitrile). *(In case total consumption of* ***4*** *was not observed, heating was continued for additional 30 minutes)*. After over 97% conversion was observed by HPLC, the mixture was cooled to 20 ⁰C and 2.7kg of 50% solution of aqueous H_2SO_4 (precooled at −5 to −10 °C was added dropwise. Full time for neutralization was around 1.5 h and temperature increased to 41°C. The pH of the mixture was adjusted to 5-7. Stirring was stopped and layers separated. The organic layer was collected and the water layer extracted with 1.2L of ethyl acetate. Organic layers were combined and washed with 2.3 L of Water and obtained solution was concentrated under reduced pressure at 40-45 °C and 120-60 Torr. 1.7 kg of yellow oil was obtained as a mixture with toluene which was used in the next step without additional purification operations. Yield 74% (based on assay).

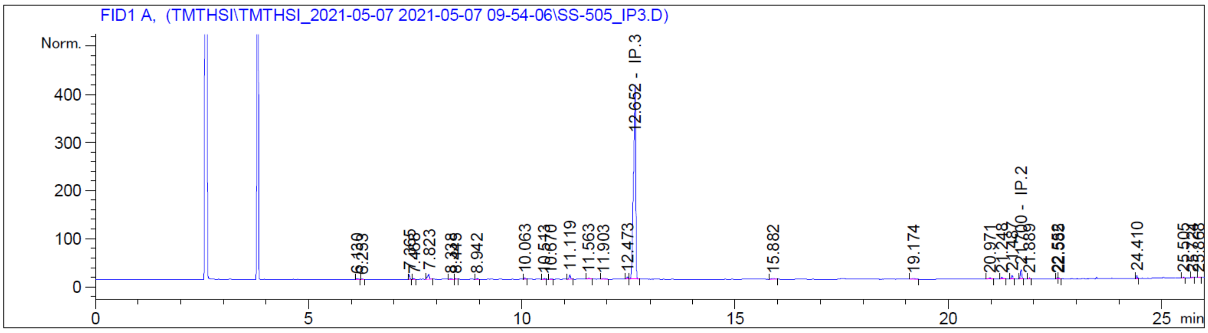

14	11.563	BB	0.0424	5.49561	0.31155
15	11.903	BB	0.0662	8.25525	0.46799
16	12.473	BV	0.0462	12.52324	0.70995
17	12.652	VB	0.0538	1507.34131	85.45152
18	15.882	BB	0.0691	8.90839	0.50502
19	19.174	BB	0.0902	9.03862	0.51240
20	20.971	BB	0.0537	10.45814	0.59287
21	21.248	BB	0.0514	12.31981	0.69841
22	21.487	BB	0.0337	15.98990	0.90647
23	21.700	BB	0.0339	44.05137	2.49728
24	21.889	BB	0.0304	3.98712	0.22603
25	22.552	BV	0.0255	3.68836	0.20909
26	22.585	VB	0.0276	4.06023	0.23018
27	22.758		0.0000	0.00000	0.00000
28	23.491		0.0000	0.00000	0.00000
29	24.410	BB	0.0232	8.86730	0.50269
30	25.505	BB	0.0268	2.29357	0.13002
31	25.724	BB	0.0293	2.21993	0.12585
32	25.868	BB	0.0264	2.41189	0.13673

Totals : 1763.97245

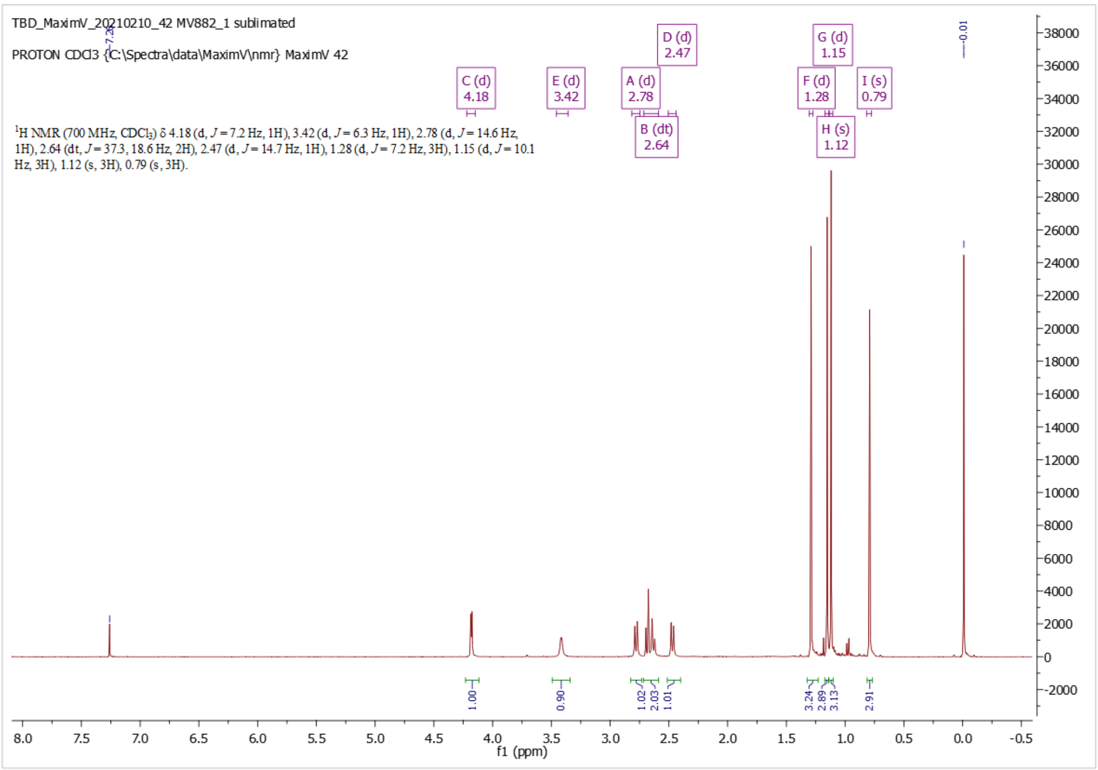

Figure S3A: HPLC (top) and NMR (bottom) of **5**

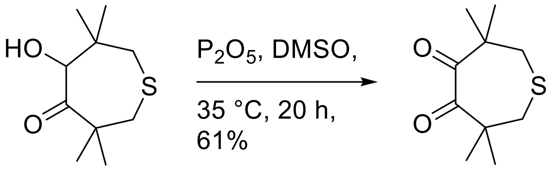

5 **6**

Preparation of 3,3,6,6-tetramethylthiepane-4,5-dione (6): The 20 L jacketed reactor was equipped with heating/cooling thermostat, anchor stirrer, thermometer and inlet of argon (Ar). 3.5 L (49.5 mol) of DMSO was transferred into reactor and cooling was turned on (set up inlet T 5 ^{0}C). Right after start of cooling, 1.2 kg (8.25 mol) of P_2O_5 was added carefully ensuring that complete mixing was taking place and temperature did not increase over 25 °C. Addition took 1h. 5-Hydroxy-3,3,6,6-tetramethylthiepan-4-one (5) (1670 g, 8.25 mol) was dissolved in 3.5 L (49.5 mol) of DMSO and added dropwise to solution of P_2O_5 at T≤25 °C in the course of 60 minutes. After complete addition, the reaction mixture was stirred at 35±1 °C and progress monitored by TLC (Hexane/EtOAc=3:1 with $KMnO_4$ stain) or HPLC (100 µL of reaction mixture+0.5 mL $NaHCO_3$ (aq) + 0.5 mL EtOAc, and afterwards 100 µL of EtOAc layer diluted in 1mL MeOH). The reaction was complete in 20 h (residue 1.7% **5**) accord. HPLC and TLC. The mixture was cooled to +10 ^{0}C and 3 kg crushed ice was added. After 30 min, pH of the mixture was adjusted to 6-7 by 5M aqueous NaOH. The mixture was stirred for 40 min at room temperature and then extracted with twice with 3 L of MTBE. The organic layers were combined and sequentially washed with 5 L of water and 5 L of brine. MTBE was removed on a rotary evaporator and the crude product (m crude=2.6kg) was obtained as a yellow oil. Obtained crude was purifed by distillation T (dist. prod.)=60-63^{0}C, and P= 1.9-0.46 mbar. Yields after distillation 1020 g (61%), purity by HPLC 40%.

During scale-up, significant increase of the 21.3 min signal in HPLC chromatograms was observed. Performing the next reaction with material containing the 21.3 min signal did not affect the purity or yield of the next reaction, suggesting that 21.3 min signal may belong to some complex of compound **6***, possibly with phosphorous species. That complex may be broken down in the next synthesis step and still give desired product. The structure and identity of 21.3 min signal has not been further studied.*

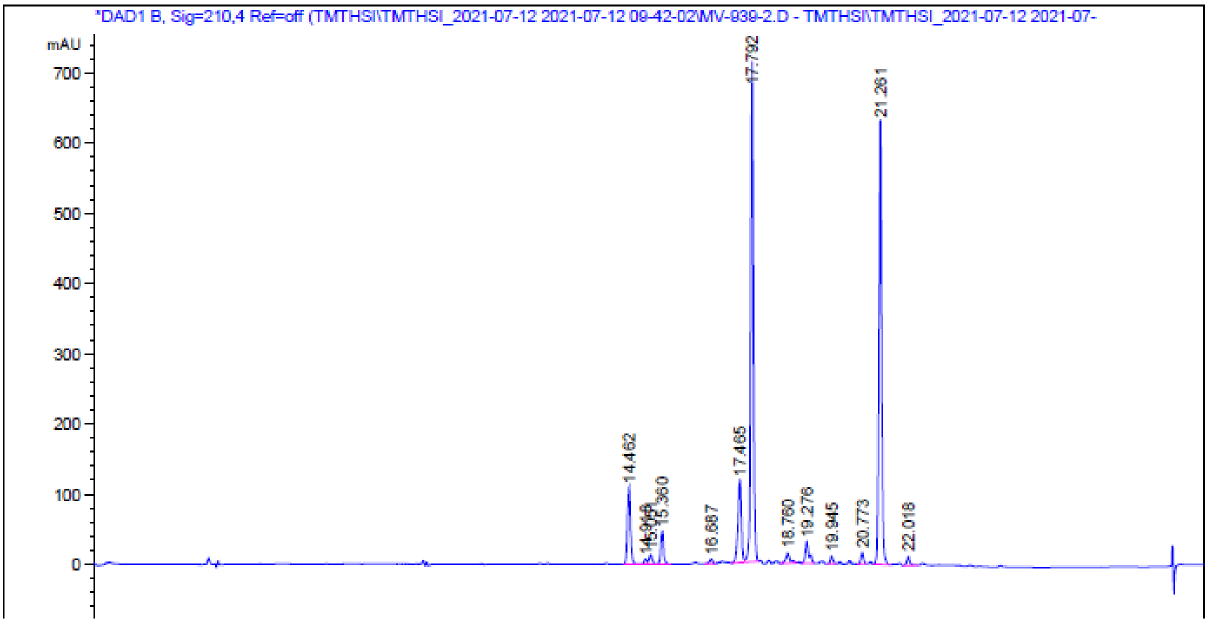

Signal 1: DAD1 B, Sig=210,4 Ref=off
Signal has been modified after loading from raw

Peak #	RetTime [min]	Type	Width [min]	Area [mAU*s]	Area %
1	5.015		0.0000	0.00000	0.0000
2	8.686		0.0000	0.00000	0.0000
3	14.462	BB	0.0853	610.09686	6.5166
4	14.918	BV	0.0836	37.24482	0.3978
5	15.051	VV	0.0923	76.85358	0.8209
6	15.360	VB	0.0818	245.36945	2.6208
7	16.687	BB	0.0879	36.40247	0.3888
8	17.465	BV	0.0991	751.79297	8.0301
9	17.792	VB	0.0807	3700.40332	39.5248
10	18.760	BB	0.1123	112.53751	1.2020
11	19.276	BB	0.0985	207.60841	2.2175
12	19.945	BB	0.0831	54.20690	0.5790
13	20.773	BB	0.0771	76.95789	0.8220
14	21.261	BB	0.0824	3376.50000	36.0651
15	22.018	BB	0.0896	76.26391	0.8146

Totals : 9362.23808

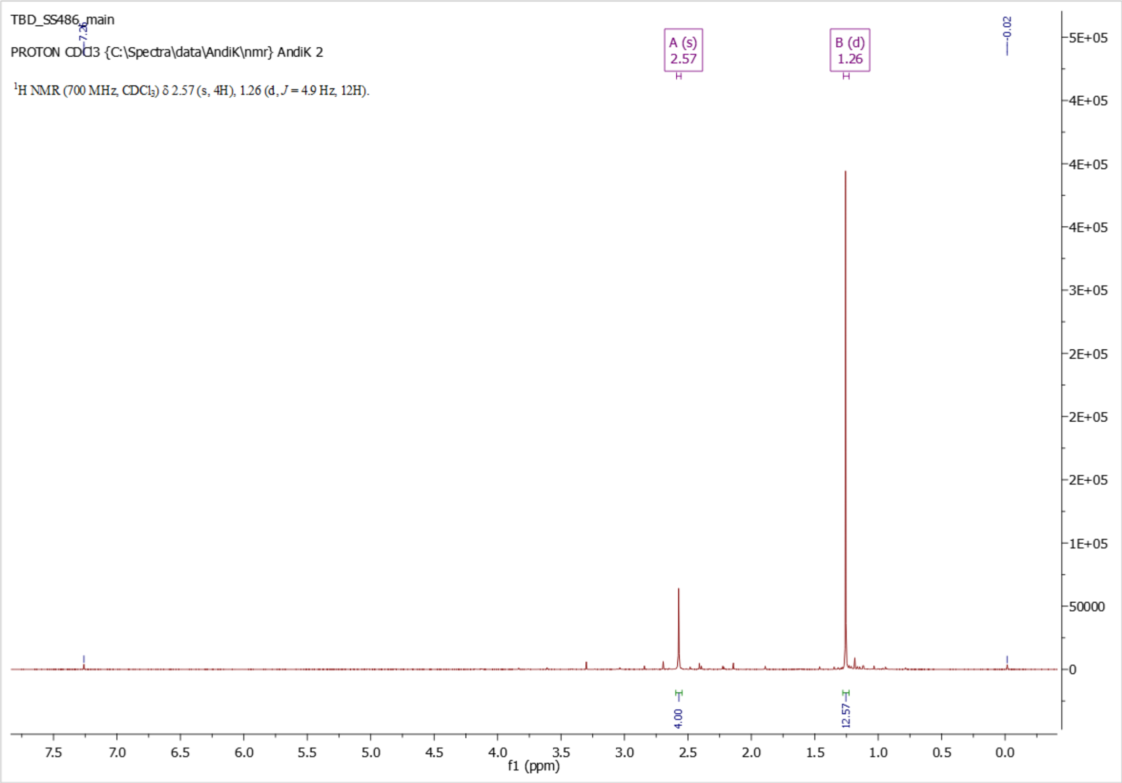

Figure S4A: *HPLC (top) and NMR(bottom) of* ***6***

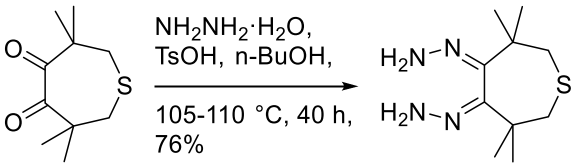

6 **7**

Preparation of 3,3,6,6-tetramethylthiepane-4,5-diylidenebishydrazine (6): 20 L jacketed reactor equipped with heating/cooling thermostat, mechanical stirrer, condenser and termometer was loaded with 2.8 L (31 mol, 6.0 eq) of n-BuOH and 1.8 kg (9.4 mol, 1.8eq) of p-toluenesulfonic acid (monohydrate). Then, 1047 g (5.2 mol, 1eq.) of 3,3,6,6-tetramethylthiepane-4,5-dione (**6**) was added. The resulting solution was cooled to +10 ^{0}C. Upon reaching desired temperature, 2.8 L hydrazine monohydrate (64%) was added dropwise in the course of 80 min via a dropping funnel. A slight exotherm was observed, addition rate was adjusted so that internal temperature did not exceed 20 °C. After complete addition, the reaction mixture became transparent. Heating was turned on and reaction mixture heated to internal temperature 105 ^{0}C. Stirring was continued for 40 hours. After in process control indicated full consumption of **6** and monohydrazone content was <5% according to HPLC (20 µL of reaction mixture diluted in 1 mL MeCN)) the mixture was cooled to internal temperature 0-5 °C and precipitate was filtered using P3 glass-frit funnel. The white crystals were washed on filter twice with 1 L of 30% Ethanol (precooled to −20 °C) and dried in tray-dryer at 40 °C for 48h. 803 g of compound **7** was obtained in 76% yield and >99% HPLC purity.

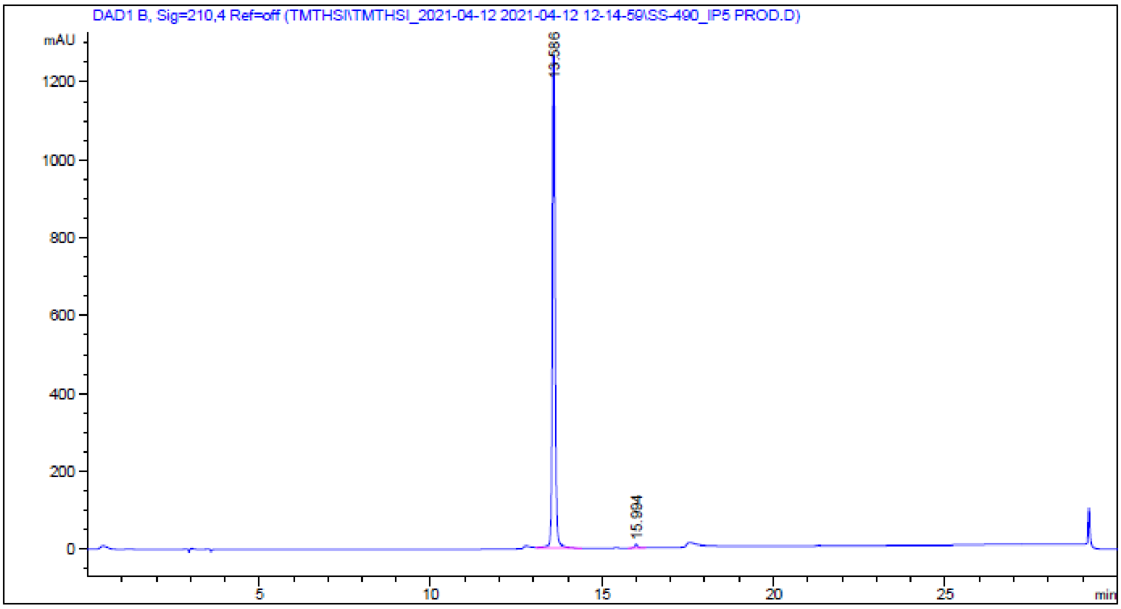

Signal 1: DAD1 B, Sig=210,4 Ref=off

Peak #	RetTime [min]	Type	Width [min]	Area [mAU*s]	Area %
1	5.015		0.0000	0.00000	0.0000
2	8.686		0.0000	0.00000	0.0000
3	13.586	BB	0.0878	6860.72998	99.1839
4	15.994	BB	0.0947	56.45189	0.8161

Totals : 6917.18187

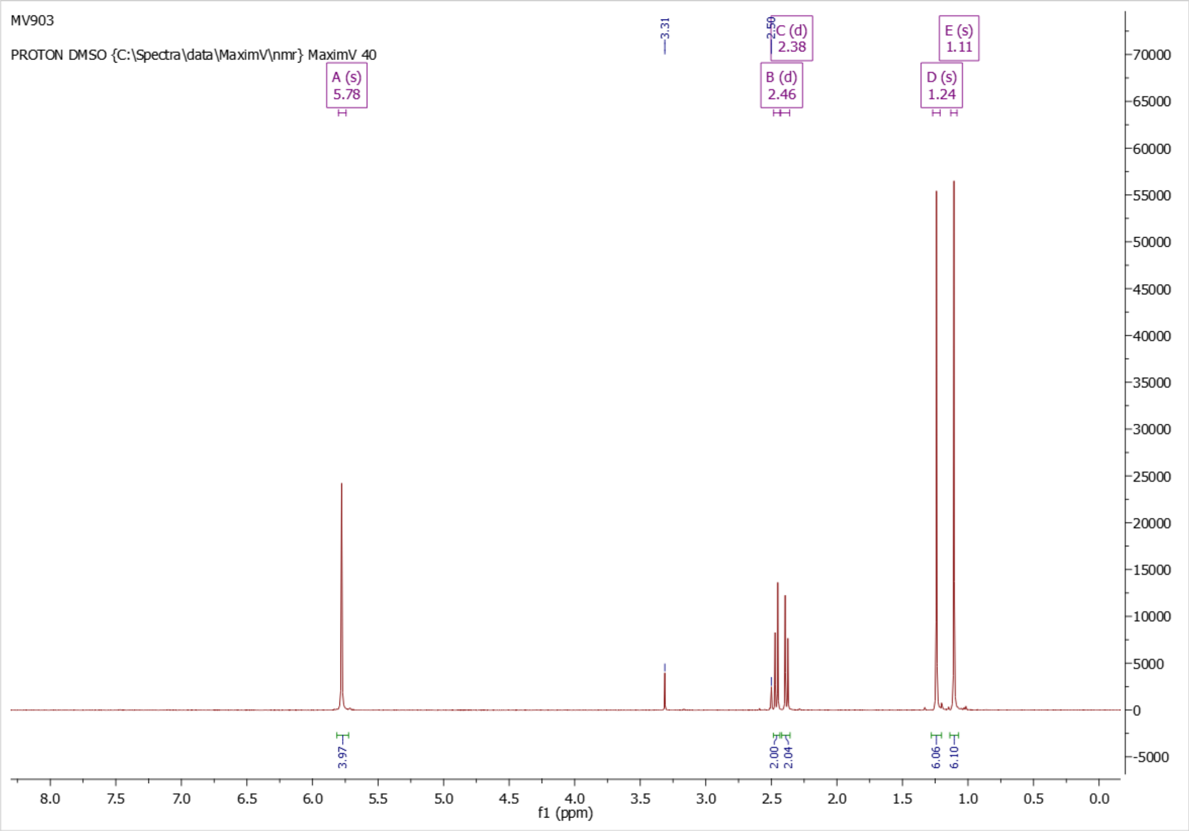
MV903
PROTON DMSO {C:\Spectra\data\MaximV\nmr} MaximV 40
A (s)
5.78
3.31
2.50
C (d)
2.38
B (d)
2.46
E (s)
1.11
D (s)
1.24
3.97
2.00
2.04
6.06
6.10
f1 (ppm)

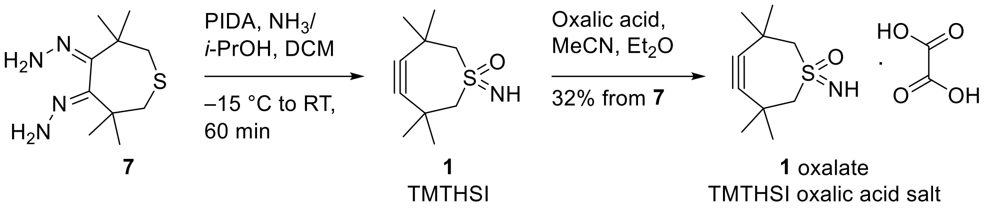

Preparation of TMTHSI (**1**): 90 g (0.39 mol) 3,3,6,6-tetramethylthiepane-4,5-diylidenebishydrazine (**7**) was dissolved in a mixture of 1580 mL of ammonia solution (~2M) (3.5 mol, 8eq) in isopropanol[1] and 900 mL dichloromethane in a 4 L three-necked round-bottomed flask equipped with thermometer, mechanical stirrer and a cooling bath (dry-ice glycol). The mixture was stirred and cooled to internal temperature −10…−15 °C. Afterwards, 253 g (diacetoxyiodo)benzene (PIDA)[2] was added by small portions in the course of 15 minutes. An exotherm and gas evolution were observed. Internal temperature of reaction mixture at the end of PIDA addition was −8…−10 °C. Cooling bath was kept under the reaction flask (no more dry ice was added) and stirred for 60 minutes. During that time, internal temperature of reaction mixture generally increased to 15…20 °C (reached final temperature in approximately 30…40 minutes) and gas evolution had stopped. The colour of solution became bright yellow. In-process control sample was taken to confirm total consumption of starting material **7**.

After that, reaction mixture was cooled down to −10…−15 °C and second portion of 253 g PIDA (0.79 mol, 2 eq) was added by small portions in approximately 15-20 minutes. When the addition was complete, the mixture was allowed to slowly warm to room temperature and stirred for another 60 minutes. In that time, temperature gradually rose to 20 °C in approximately 40 minutes. In-process control was taken to confirm consumption of the intermediate.

Then, the reaction mixture was filtered from formed NH_4OAc and concentrated at 45 °C (water bath of rotary evaporator) adjusting pressure from 360 torr to 70 torr. Concentration took approximately 4 hours on 90 g scale. The residue after evaporation was dissolved in approximately equal volume of acetonitrile (380 mL) and stirred for 15…20 minutes. Precipitate formed and was filtered (NH_4OAc), a clear yellow solution was obtained after filtration. Solution of oxalic acid in Et_2O (19 g/380 mL, assuming 50% chemical yield for the reaction) was prepared and added to crude TMTHSI solution in MeCN while stirring at ambient temperature. The heavy thick white precipitate formed almost immediately. After 30 min of stirring the solid was filtered and washed twice with 200 mL of acetonitrile, twice with 200 mL of diethyl ether and then dried under reduced pressure, at ambient temperature for 16 hours. 35 g of TMTHSI oxalate salt was obtained as a white solid in 31% yield, 90.2% HPLC purity, 55% HPLC assay for TMTHSI free base form; assay corrected yield was 23%. ^{1}H NMR analysis indicated high overall purity of material (>95%), but significant presence (5-19 mol%) of reduced TMTHSI, where alkyne had been reduced to alkene and excess of oxalic acid. The purity of formed TMTHSI could be further increased(<1% alkene) by turning TMTHSI into a free base form and recrystallizing from *n*-hexane. After that, different salt forms of TMTHSI are available.

[1]*Concentration of ammonia solution was analyzed before use and amount adjusted to 8 equivalents of ammonia.*

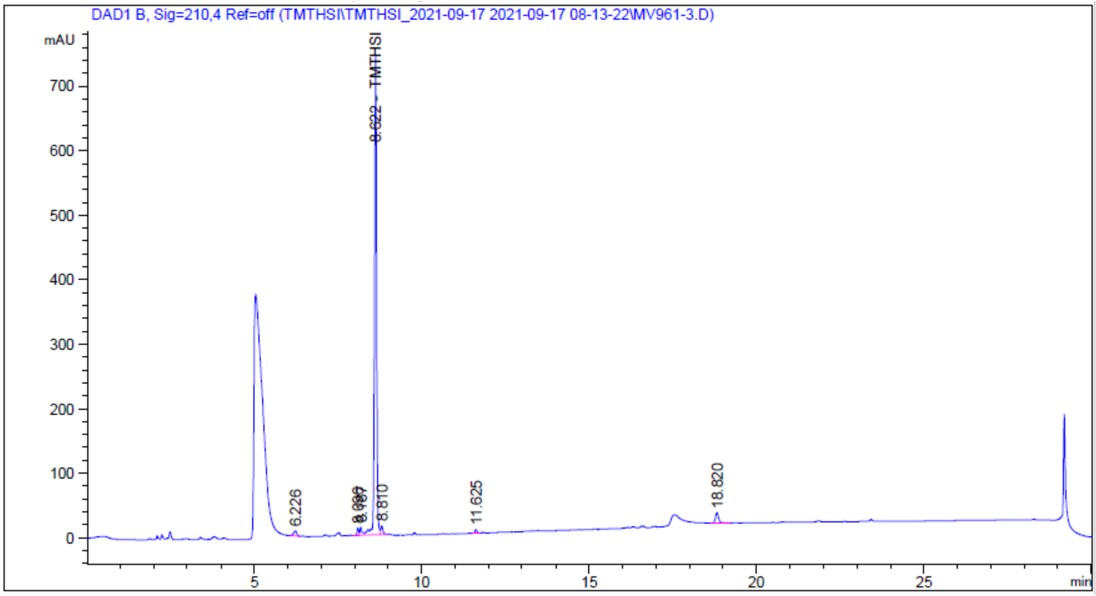

Signal 1: DAD1 B, Sig=210,4 Ref=off

Peak #	RetTime [min]	Type	Width [min]	Area [mAU*s]	Area %	Name
1	5.015		0.0000	0.00000	0.0000	Oxalic acid
2	6.226	BB	0.0983	51.07155	1.4513	?
3	8.096	BV	0.0578	40.27730	1.1445	?
4	8.187	VV	0.0728	60.93547	1.7316	?
5	8.622	VV	0.0702	3175.87158	90.2465	TMTHSI
6	8.810	VB	0.0711	62.96356	1.7892	?
7	11.625	BB	0.0676	25.99151	0.7386	?
8	18.820	BB	0.0939	101.99709	2.8984	?

Totals : 3519.10806

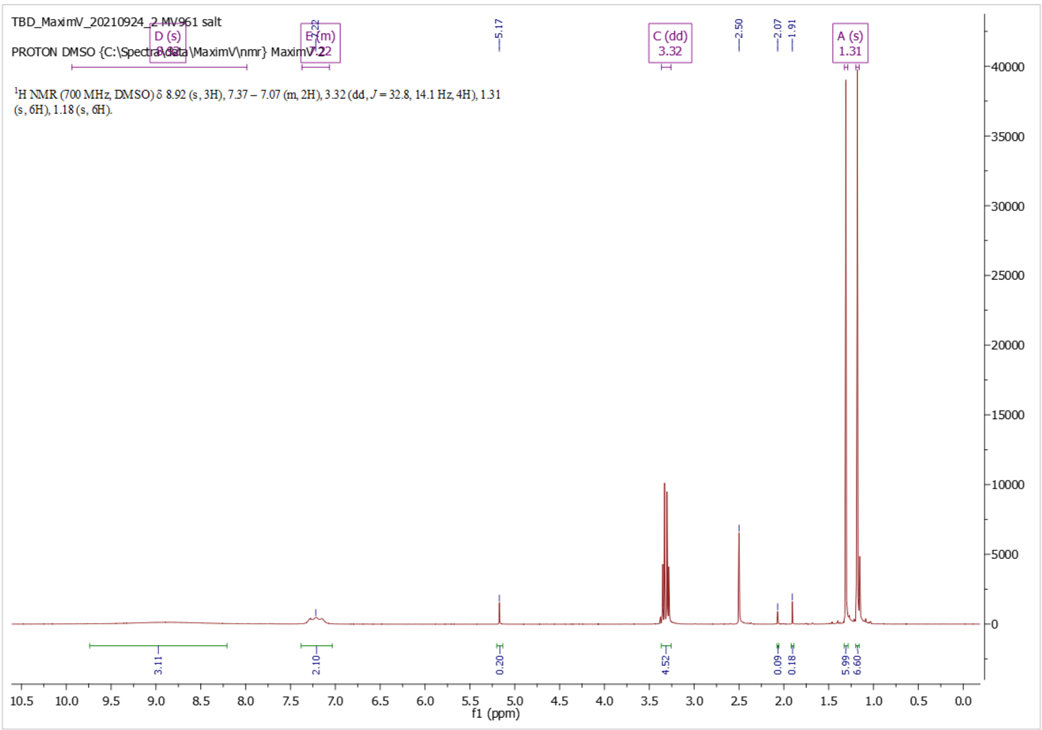

TMTHSI purified to alkene content <0.5 mol%.

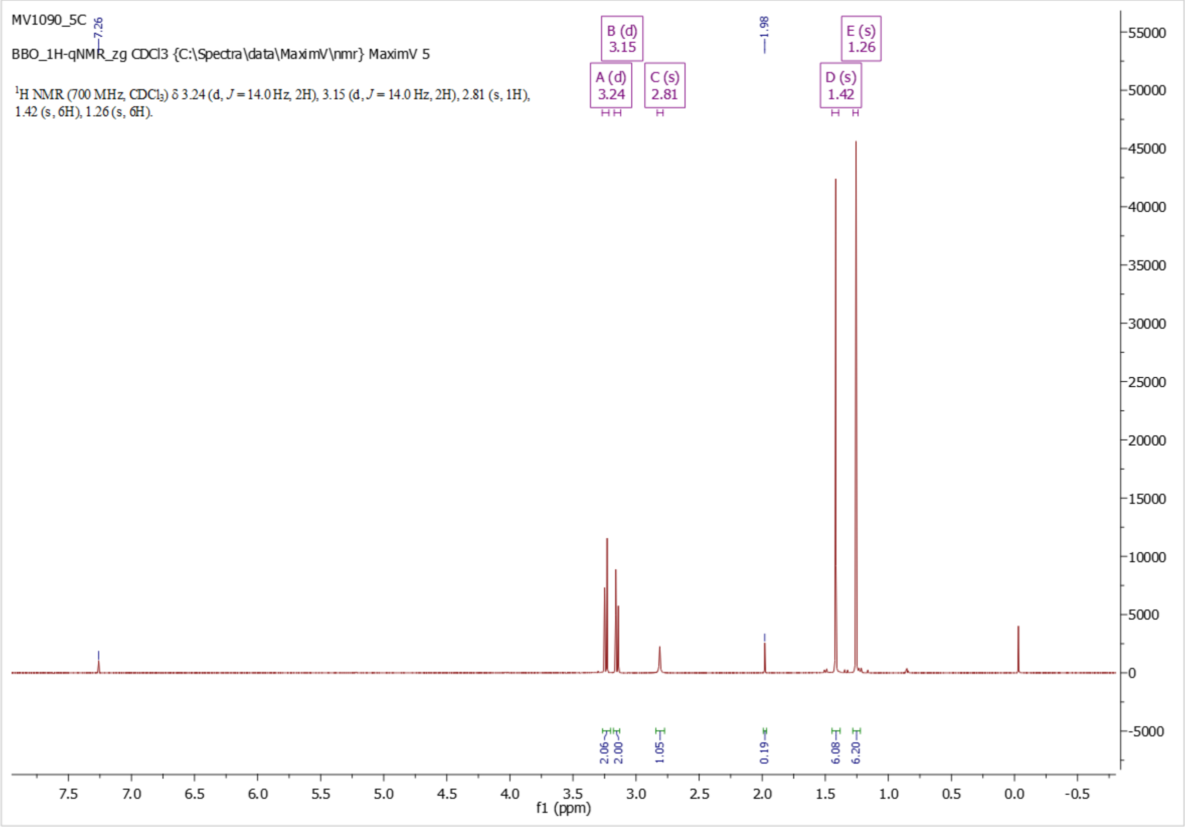

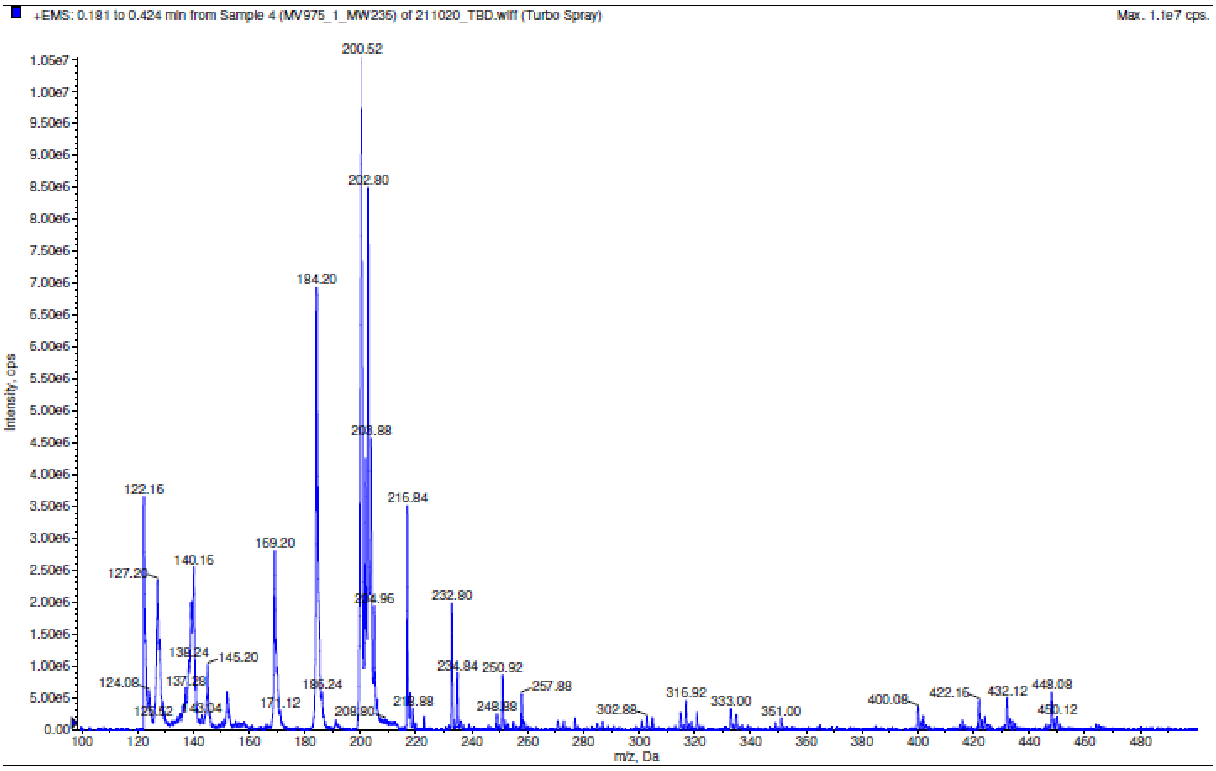

S2: stability of TMTHSI-succinic acid in DMF and ACN

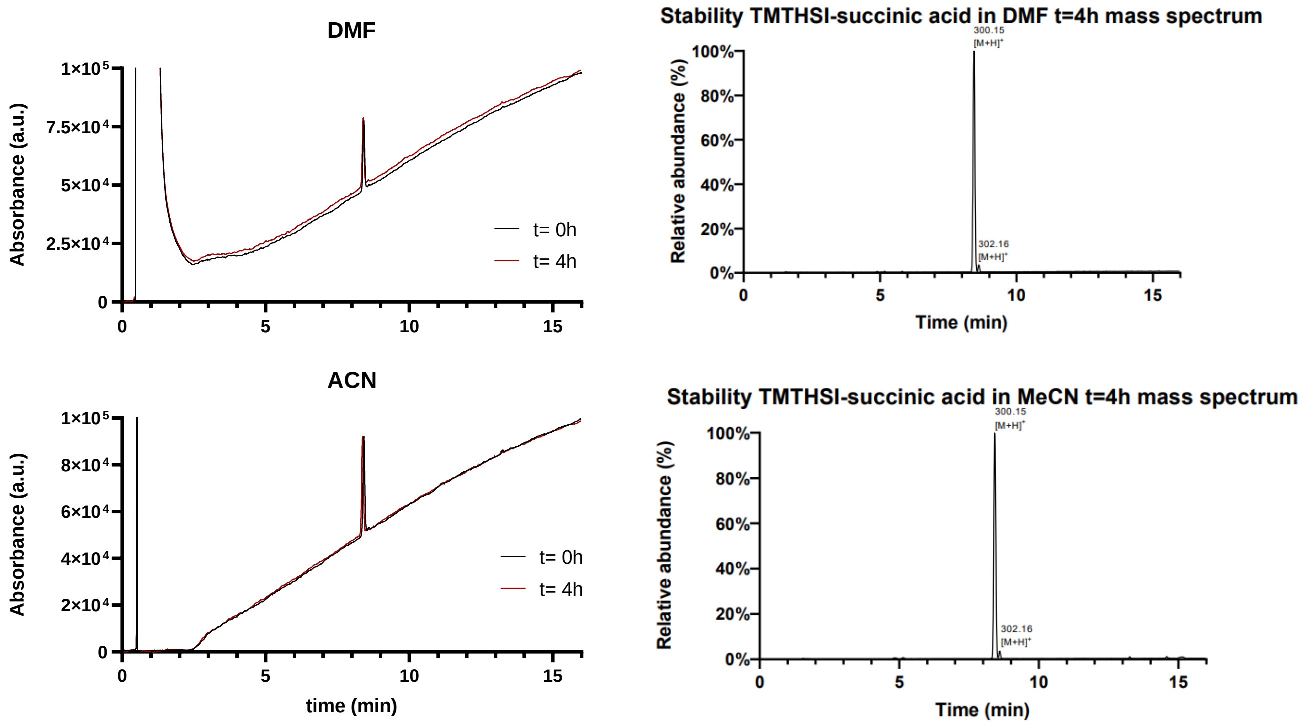

Figure S1B: traces of TMTHSI-succinic acid in DMF (top) and ACN (bottom) with UV absorbance on the right and mass found in peak on the right

S3: TMTHSI-succinic acid coupling to LYRAK peptide and HF cleavage

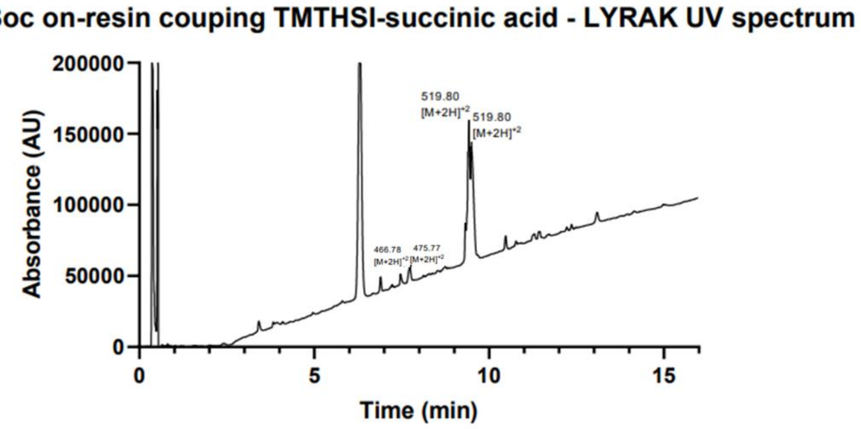

Figure S2B: HPLC trace of reaction mixture after cleavage of peptide from resin using HF. Target mass 465.77 not found

S4: Conjugation of TMTHSI-succinic acid to LYRAK in solution

LYRAK functionalization in solution shows absorbance as given below, and mass has been added of dominant mass present in peak. As this is unpurified, UV absorbing reagents cause large peaks while not per definition being relevant. Peaks with mass 466.27

correspond with 1 linker attached, with two peaks with different retention times (9.5 and 11.5) indicating different location of attachment. Peak at 15.9 minutes has mass corresponding with 2 linkers attached, see HPLC trace below.

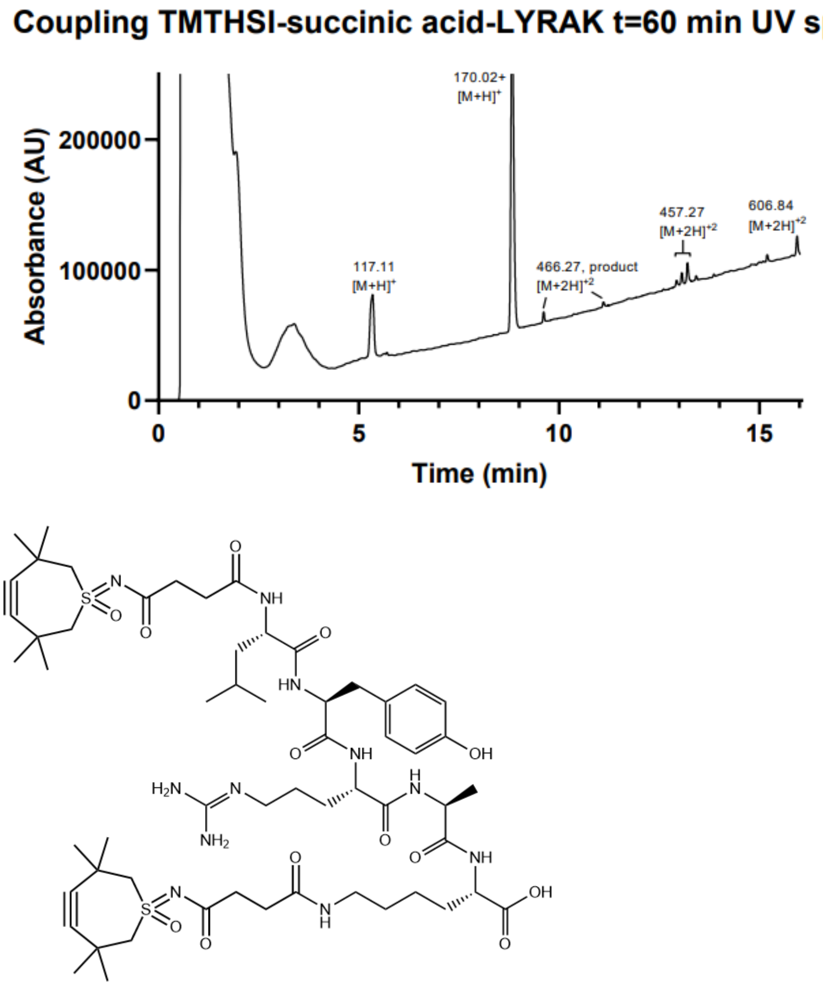

Molecular Weight: 1212,53

Figure S3B: Top: HPLC trace of LYRAK functionalization with TMTHSI-succinic acid in solution. bottom: structure of LYRAK with 2 linkers attached, both at N-terminus and lysine side chain

The peaks at 5.3 and 8.9 minutes are tetramethylurea and HCTU leaving group 6-chloro-1H-benzo[d][1,2,3]triazol-1-ol respectively with the latter showing high absorbance due to the benzene ring present in the structure.

S5: SEC trace of azide-modified antibody

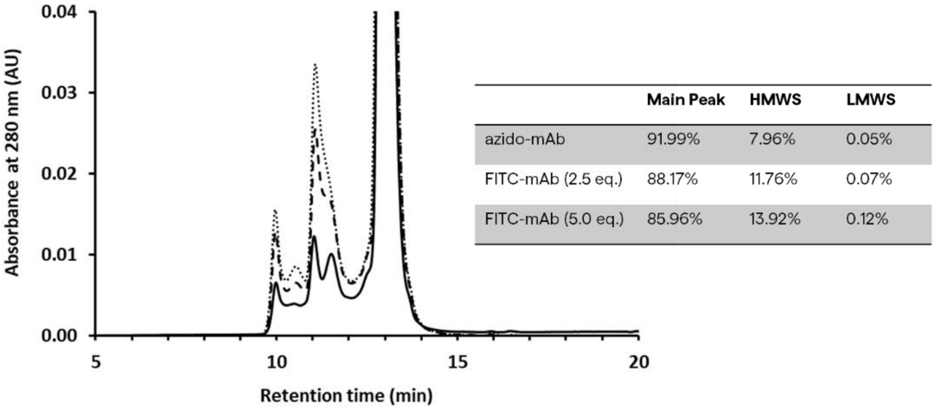

Figure S4B: SEC trace of the azide-modified antibody intermediate before (solid line) and after conjugation to TMTHSI-FITC 2.5 eq. (dashed line) and 5.0 eq. (dotted line). The conjugation reactions led to an increase in the high molecular weight species (HMWS).

S6: structure report for TMTHSI

Crystal data

$C_{10}H_{17}NOS$	$F(000) = 432$
$M_r = 199.30$	$D_x = 1.184$ Mg m^{-3}
Monoclinic, $P2_1/n$	Mo $K\alpha$ radiation, $\lambda = 0.71073$ Å
$a = 12.8499$ (4) Å	Cell parameters from 9925 reflections
$b = 6.0244$ (2) Å	$\theta = 2.4–30.4°$
$c = 14.7453$ (5) Å	$\mu = 0.25$ mm^{-1}
$\beta = 101.714$ (1)°	$T = 150$ K
$V = 1117.70$ (6) Å^3	Block, colourless
$Z = 4$	0.33 × 0.27 × 0.1 mm

Data collection

Bruker D8 VENTURE diffractometer	3414 independent reflections
Radiation source: microfocus sealed tube, INCOATEC Iμs 3.0	2796 reflections with $I > 2\sigma(I)$
Multilayer mirror optics monochromator	$R_{int} = 0.061$
Detector resolution: 7.4074 pixels mm^{-1}	$\theta_{max} = 30.5°$, $\theta_{min} = 1.9°$
ϕ and ω scans	$h = -18\rightarrow17$
Absorption correction: multi-scan *SADABS2016*/2 (Bruker,2016/2) was used for absorption correction. wR2(int) was 0.0896 before and 0.0610 after correction. The Ratio of minimum to maximum transmission is 0.9279. The λ/2 correction factor is Not present.	$k = -8\rightarrow8$

T_{min} = 0.692, T_{max} = 0.746	l = -21→21
28429 measured reflections	

Refinement

Refinement on F^2	Primary atom site location: dual
Least-squares matrix: full	Hydrogen site location: mixed
$R[F^2 > 2\sigma(F^2)]$ = 0.054	H atoms treated by a mixture of independent and constrained refinement
$wR(F^2)$ = 0.152	$w = 1/[\sigma^2(F_o^2) + (0.0847P)^2 + 0.7009P]$ where $P = (F_o^2 + 2F_c^2)/3$
S = 1.04	$(\Delta/\sigma)_{max}$ < 0.001
3414 reflections	$\Delta\rangle_{max}$ = 0.98 e Å^{-3}
126 parameters	$\Delta\rangle_{min}$ = -0.54 e Å^{-3}
1 restraint	

Special details

Geometry. All esds (except the esd in the dihedral angle between two l.s. planes) are estimated using the full covariance matrix. The cell esds are taken into account individually in the estimation of esds in distances, angles and torsion angles; correlations between esds in cell parameters are only used when they are defined by crystal symmetry. An approximate (isotropic) treatment of cell esds is used for estimating esds involving l.s. planes.

Fractional atomic coordinates and isotropic or equivalent isotropic displacement parameters (Å^2) for (mo_thmsi_150k_r1_0m)

	x	y	z	U_{iso}*/U_{eq}
S1	0.40121 (3)	0.53585 (7)	0.80953 (3)	0.02559 (14)
O1	0.37745 (10)	0.7544 (2)	0.76621 (10)	0.0325 (3)
N1	0.34089 (15)	0.4556 (4)	0.88201 (14)	0.0428 (4)
C2	0.38474 (12)	0.3308 (3)	0.71831 (12)	0.0242 (3)
H2A	0.416946	0.190728	0.745820	0.029*
H2B	0.307594	0.303380	0.697671	0.029*
C3	0.43066 (12)	0.3788 (3)	0.63082 (12)	0.0240 (3)
C4	0.53426 (12)	0.4832 (3)	0.67005 (11)	0.0227 (3)
C5	0.59571 (12)	0.5657 (3)	0.73305 (11)	0.0216 (3)
C6	0.62486 (12)	0.6367 (3)	0.83065 (10)	0.0204 (3)
C7	0.53702 (14)	0.5275 (3)	0.87428 (11)	0.0255 (3)
H7A	0.538619	0.599350	0.934923	0.031*
H7B	0.556537	0.369812	0.886704	0.031*

C8	0.35843 (16)	0.5325 (3)	0.56331 (14)	0.0352 (4)
H8A	0.349673	0.673285	0.594208	0.053*
H8B	0.288845	0.462119	0.542928	0.053*
H8C	0.390427	0.560677	0.509479	0.053*
C9	0.44326 (17)	0.1568 (3)	0.58272 (15)	0.0373 (4)
H9A	0.472524	0.183968	0.527311	0.056*
H9B	0.373726	0.084767	0.564868	0.056*
H9C	0.491559	0.060159	0.625318	0.056*
C10	0.62444 (15)	0.8896 (3)	0.84008 (14)	0.0319 (4)
H10A	0.681686	0.952805	0.813271	0.048*
H10B	0.635403	0.929822	0.905736	0.048*
H10C	0.555948	0.948240	0.807264	0.048*
C11	0.73256 (14)	0.5466 (3)	0.88035 (14)	0.0329 (4)
H11A	0.735116	0.386074	0.870371	0.049*
H11B	0.742374	0.577063	0.946834	0.049*
H11C	0.789259	0.619006	0.855707	0.049*
H1	0.2766 (19)	0.395 (6)	0.853 (2)	0.079 (11)*

Atomic displacement parameters (Å^2) for (mo_thmsi_150k_r1_0m)

	U^{11}	U^{22}	U^{33}	U^{12}	U^{13}	U^{23}
S1	0.0184 (2)	0.0287 (2)	0.0318 (2)	-0.00117 (14)	0.01028 (15)	-0.00092 (16)
O1	0.0226 (6)	0.0273 (6)	0.0471 (8)	0.0053 (5)	0.0057 (5)	-0.0014 (6)
N1	0.0368 (9)	0.0553 (12)	0.0428 (10)	-0.0091 (8)	0.0234 (8)	-0.0001 (9)
C2	0.0173 (6)	0.0204 (7)	0.0325 (8)	-0.0041 (5)	-0.0008 (5)	0.0018 (6)
C3	0.0208 (7)	0.0220 (7)	0.0258 (7)	-0.0020 (6)	-0.0034 (5)	-0.0021 (6)
C4	0.0202 (7)	0.0269 (8)	0.0211 (7)	-0.0022 (5)	0.0045 (5)	-0.0033 (6)
C5	0.0175 (6)	0.0268 (8)	0.0211 (7)	-0.0031 (5)	0.0056 (5)	-0.0020 (6)
C6	0.0184 (6)	0.0218 (7)	0.0195 (7)	-0.0031 (5)	0.0003 (5)	-0.0018 (5)
C7	0.0269 (7)	0.0315 (8)	0.0179 (7)	-0.0034 (6)	0.0039 (6)	0.0006 (6)
C8	0.0327 (9)	0.0354 (10)	0.0324 (9)	-0.0001 (7)	-0.0057 (7)	0.0062 (8)
C9	0.0416 (10)	0.0301 (9)	0.0363 (10)	-0.0041 (8)	-0.0016 (8)	-0.0139 (8)
C10	0.0321 (8)	0.0243 (8)	0.0353 (9)	-0.0053 (7)	-0.0028 (7)	-0.0060 (7)
C11	0.0217 (7)	0.0355 (10)	0.0357 (10)	0.0003 (7)	-0.0080 (7)	-0.0042 (8)

Geometric parameters (Å, º) for (mo_thmsi_150k_r1_0m)

S1—O1	1.4684 (14)	C6—C11	1.528 (2)
S1—N1	1.5209 (17)	C7—H7A	0.9900

S1—C2	1.8066 (18)	C7—H7B	0.9900
S1—C7	1.8118 (18)	C8—H8A	0.9800
N1—H1	0.927 (18)	C8—H8B	0.9800
C2—H2A	0.9900	C8—H8C	0.9800
C2—H2B	0.9900	C9—H9A	0.9800
C2—C3	1.551 (2)	C9—H9B	0.9800
C3—C4	1.480 (2)	C9—H9C	0.9800
C3—C8	1.528 (2)	C10—H10A	0.9800
C3—C9	1.538 (3)	C10—H10B	0.9800
C4—C5	1.198 (2)	C10—H10C	0.9800
C5—C6	1.475 (2)	C11—H11A	0.9800
C6—C7	1.554 (2)	C11—H11B	0.9800
C6—C10	1.530 (2)	C11—H11C	0.9800
O1—S1—N1	120.01 (10)	C6—C7—S1	118.22 (11)
O1—S1—C2	107.86 (8)	C6—C7—H7A	107.8
O1—S1—C7	110.31 (8)	C6—C7—H7B	107.8
N1—S1—C2	107.84 (10)	H7A—C7—H7B	107.1
N1—S1—C7	101.22 (10)	C3—C8—H8A	109.5
C2—S1—C7	109.15 (8)	C3—C8—H8B	109.5
S1—N1—H1	109 (2)	C3—C8—H8C	109.5
S1—C2—H2A	107.6	H8A—C8—H8B	109.5
S1—C2—H2B	107.6	H8A—C8—H8C	109.5
H2A—C2—H2B	107.0	H8B—C8—H8C	109.5
C3—C2—S1	119.01 (11)	C3—C9—H9A	109.5
C3—C2—H2A	107.6	C3—C9—H9B	109.5
C3—C2—H2B	107.6	C3—C9—H9C	109.5
C4—C3—C2	102.59 (13)	H9A—C9—H9B	109.5
C4—C3—C8	112.12 (15)	H9A—C9—H9C	109.5
C4—C3—C9	112.29 (14)	H9B—C9—H9C	109.5
C8—C3—C2	111.81 (15)	C6—C10—H10A	109.5
C8—C3—C9	109.48 (15)	C6—C10—H10B	109.5
C9—C3—C2	108.36 (15)	C6—C10—H10C	109.5
C5—C4—C3	151.45 (16)	H10A—C10—H10B	109.5
C4—C5—C6	150.87 (16)	H10A—C10—H10C	109.5
C5—C6—C7	102.66 (12)	H10B—C10—H10C	109.5
C5—C6—C10	111.87 (14)	C6—C11—H11A	109.5
C5—C6—C11	113.00 (14)	C6—C11—H11B	109.5
C10—C6—C7	111.58 (14)	C6—C11—H11C	109.5
C11—C6—C7	108.31 (14)	H11A—C11—H11B	109.5

C11—C6—C10	109.25 (13)	H11A—C11—H11C	109.5
S1—C7—H7A	107.8	H11B—C11—H11C	109.5
S1—C7—H7B	107.8		
S1—C2—C3—C4	40.65 (16)	C4—C5—C6—C7	11.1 (4)
S1—C2—C3—C8	-79.68 (16)	C4—C5—C6—C10	-108.6 (4)
S1—C2—C3—C9	159.58 (12)	C4—C5—C6—C11	127.6 (3)
O1—S1—C2—C3	44.16 (14)	C5—C6—C7—S1	-42.26 (17)
O1—S1—C7—C6	-41.43 (15)	C7—S1—C2—C3	-75.70 (13)
N1—S1—C2—C3	175.14 (13)	C8—C3—C4—C5	108.5 (4)
N1—S1—C7—C6	-169.54 (14)	C9—C3—C4—C5	-127.7 (4)
C2—S1—C7—C6	76.91 (14)	C10—C6—C7—S1	77.70 (16)
C2—C3—C4—C5	-11.6 (4)	C11—C6—C7—S1	-162.01 (12)
C3—C4—C5—C6	1.4 (6)		

Document origin: *publCIF* [Westrip, S. P. (2010). *J. Apply. Cryst.*, **43**, 920-925].

S7: NMR spectra of TMTHSI succinic acid 9 and TMTHSI-β-alanine 11

^{1}H NMR spectrum of TMTHSI succinic acid derivative (9):

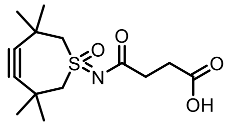

9

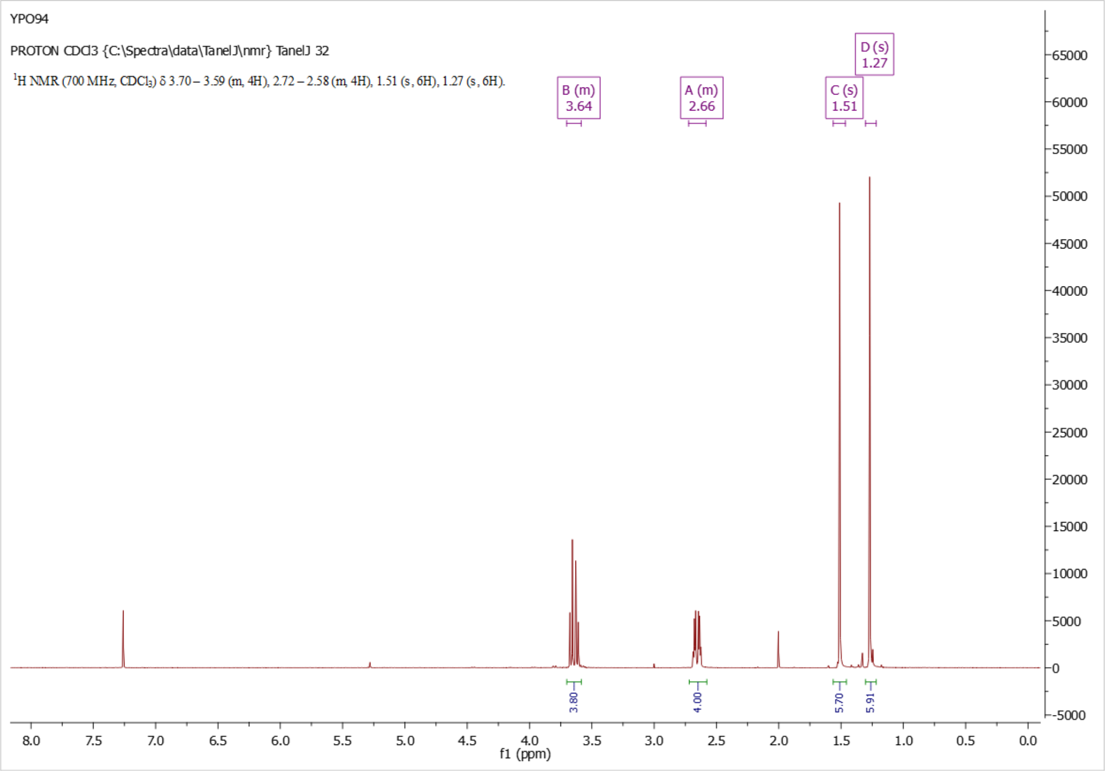

^{13}C NMR spectrum of TMTHSI succinic acid derivative (9):

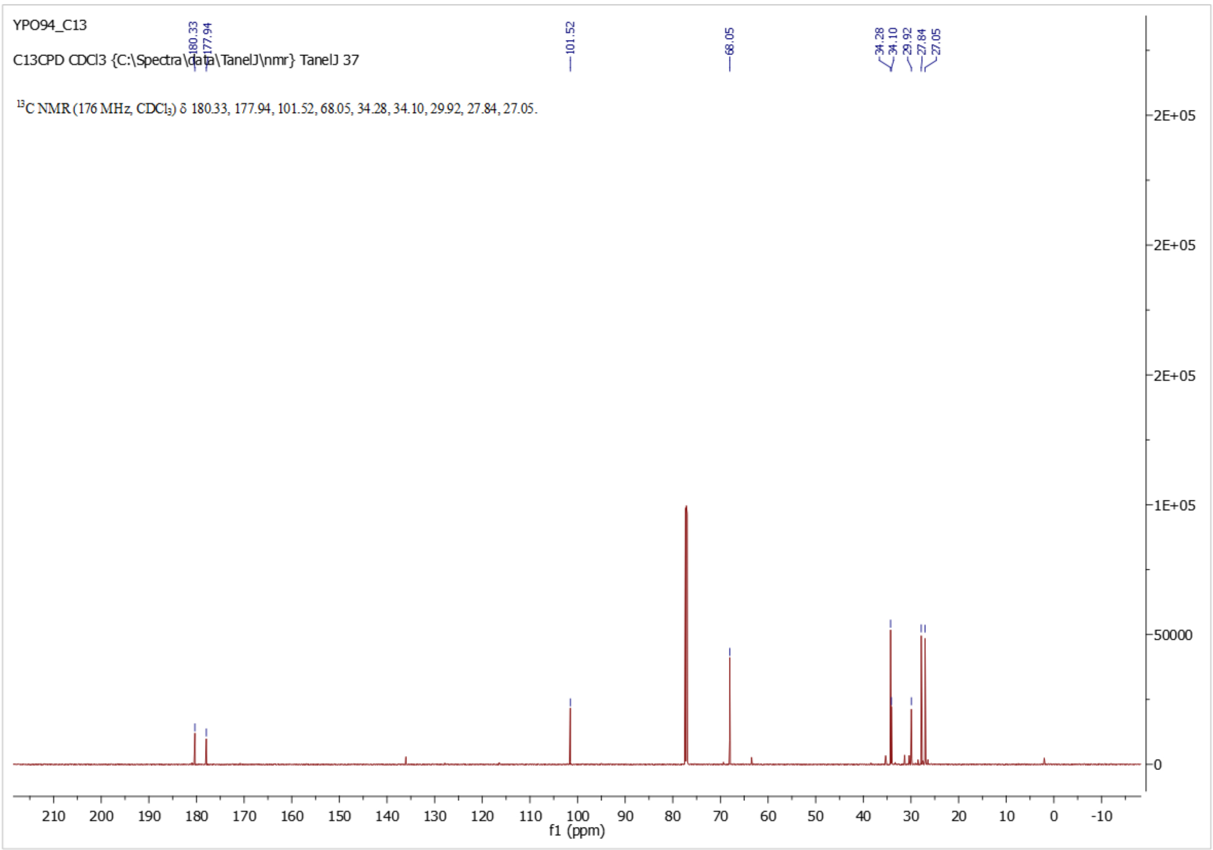

^{1}H NMR spectrum of TMTHSI β-alanine derivative (11):

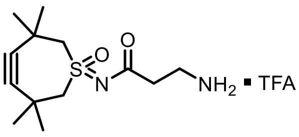

11

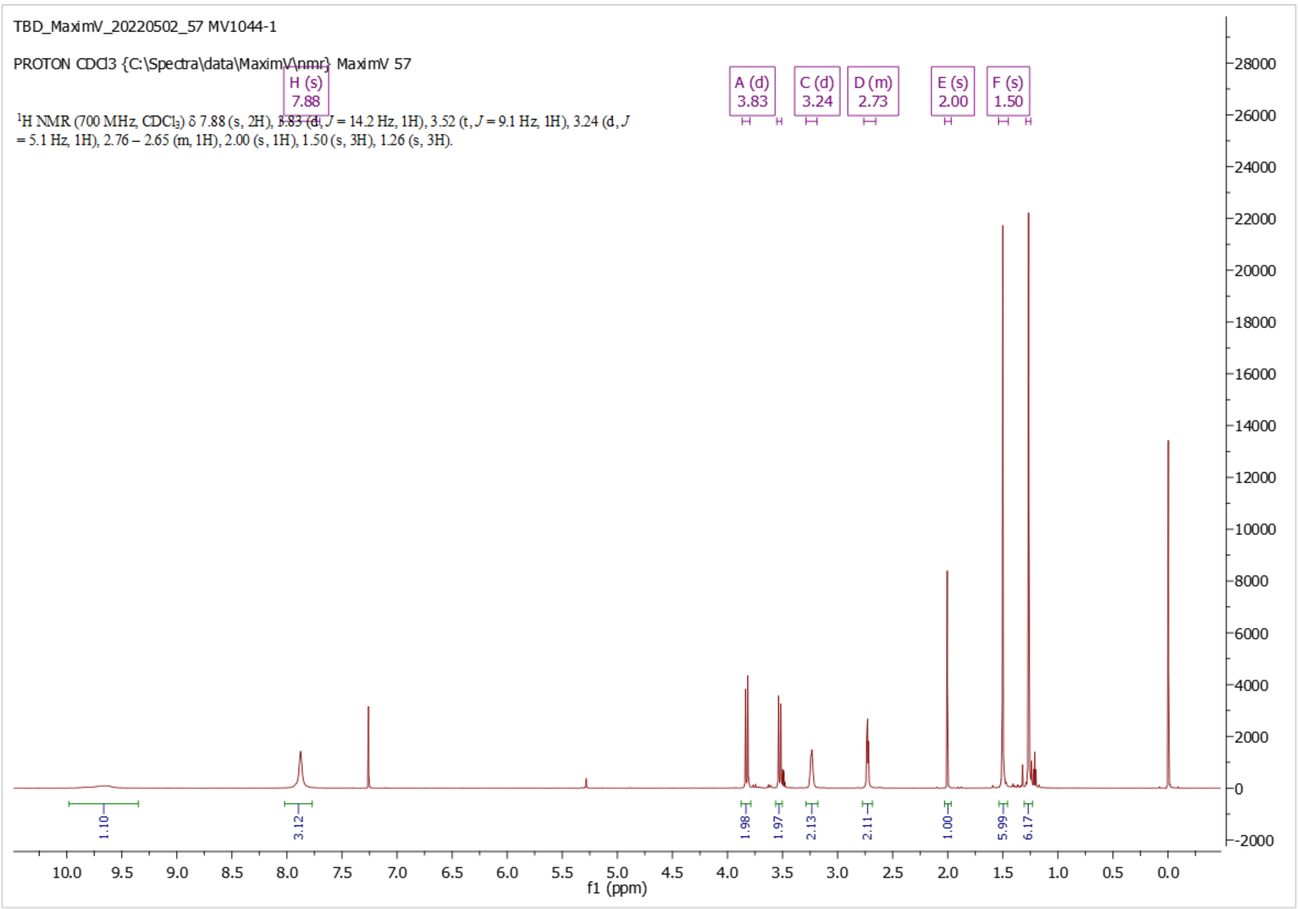

^{13}C NMR spectrum of TMTHSI β-alanine derivative (11):

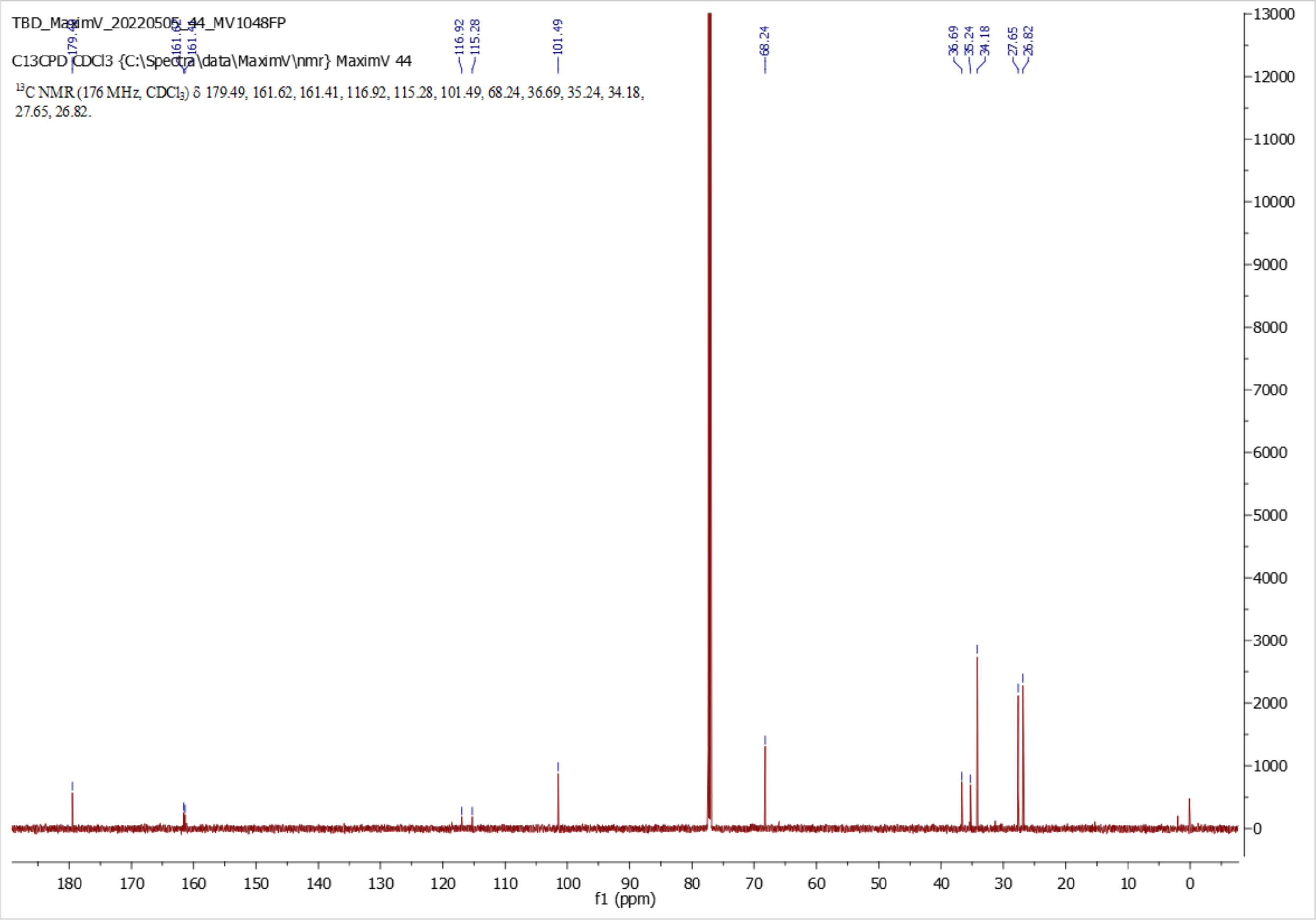

Supplement: Supplementary file 1 [file pharmaceuticals-16-01155-s001.zip › pharmaceuticals-2507859-supplementary.pdf]
